# Supplementary material for: Interventions for subjective cognitive decline: systematic review and meta-analysis
Source: BMJ Open. 2018 Jul 19;8(7):e021610. doi: 10.1136/bmjopen-2018-021610 (PMC6059327; doi:10.1136/bmjopen-2018-021610)
Supplement: Supplementary file 2 [file bmjopen-2018-021610supp002.pdf]

Table DS1. Characteristics and validity of included studies

Study	Recruitment Setting	Inclusion criteria	Treatment group		Control group		Outcomes	Key findings: treatment vs control group, measured at end of intervention unless stated	Validity (answer to questions 1-5 in methods)				
			Treatment	n	Treatment	n			1	2	3	4	5
*Andrewes et al	Volunteers in the community	SMC; MDRS score > 123; Age 60-70 years	Memory training- participants provided with a handbook containing information and practice exercises to aid memory.<br><br>4 weeks	20	Non-active control- pamphlets providing information about three mnemonics.<br><br>4 weeks	20	Memory strategy knowledge questionnaire, Memory Strategy Use Questionnaire, memory diary.<br><br>Face-Name test, Prospective memory,	No differences in prospective memory tasks.<br><br>Significant improvement in face-name recall in memory training group.<br><br>No difference in cognitive measures at 4-months follow up.	y	y	y	y	n
Barnes et al	Volunteers in the community	SMC; Age >65 years	Mental activity performed on computers to enhance auditory and visual processing.<br><br>60 mins, 3 days a week for 12 weeks.	32	Active control- watched DVDs of educational lectures, on art, history and science.<br><br>60 mins, 3 days a week for 12 weeks	31	RAVLT, EFT, DSST, TMT-A, TMT-B, UFOV, Verbal fluency	Visuospatial function improved significantly for treatment group; improved global cognitive functioning in both groups but no significant inter-group differences.	y	y	y	y	y

Barnes et al	Volunteers in the community	SMC; Age>65 years	Aerobic exercise aiming to increase heart rates to 60-75% of participants' maximum.<br><br>60 mins, 3 days a week for 12 weeks	32	Active control- without aerobic exercise portion. Aim was not to increase resting heart rate-<br><br>60 mins, 3 days a week for 12 weeks	31	RAVLT, EFT, DSST, TMT-A, TMT-B, UFOV, Verbal fluency	Improved global cognitive functioning in both groups but no significant inter-group differences.	y	y	y	y	y
Beck et al	Volunteers in the community	SMI as diagnosed by the PRMQ.	Gingko Biloba 240mg OD<br><br>8 weeks	43	Placebo<br><br>8 weeks	32	Task set shifting, delayed response task, prospective memory task, Go-NoGo task,<br><br>MDBF subscales for good/bad mood; alertness/fatigue; calmness/restlessness pre and post TSST	Significant improvement in task-set shifting (executive function) in treatment group.	y	y	y	y	n
Bosepflug et al	Volunteers in the community	SMC	Fish oil (1.6g eicosapentaenoic acid (EPA) and 0.8g	11	Placebo- corn oil<br><br>24 weeks	10	Working memory task	Significant improvement of working memory in the 2-back	y	y	_	y	n

			docosahexaenoic acid (DHA)) daily.<br><br>24 weeks.					condition. In the treatment group					
Brautigham et al	Volunteers in the community	SCD; non-institutionalised; Age 55-86 years	Gingko 40 drops (1.9mls) undiluted daily<br><br>24 weeks	72	Placebo<br><br>24 weeks	82	15 Words of Rey (part 1 and 2); Benton test of visual retention-revised; EMCT	Statistically significant improvement in visual memory that was dose-dependent	y	y	n	y	n
Brautigham et al	Volunteers in the community	SCD;<br>non-institutionalised;<br>Age 55-86 years	Gingko 40 drops (1.9mls) 1:1 dilution<br><br>24 weeks	72	Placebo<br><br>24 weeks	82	15 Words of Rey (part 1 and 2); Benton test of visual retention-revised; EMCT	Statistically significant improvement in visual memory that was dose-dependent	y	y	n	y	n
Cohen-Mansfield et al	Volunteers in the community	SMC<br>MMSE >24;<br>Hebrew language;<br>Age>65 years	Cogntive training course- participant centred, delivered in a book club format with memory, reasoning and speed of processing being the main foci.	15	Waiting list control	28	UCLA loneliness scale<br><br>Global cognitive score;	Significantly improved global cognitive score and significant reduction in loneliness and self-reported memory difficulties.	y	y	n	y	n

			10 weeks										
Cohen-Mansfield et al	Volunteers in the community	SMC MMSE >24; Hebrew language; Age>65 years	Participant-centered course-delivery of memory, cognitive and organisational strategies. Cognitive behavioural principles used whereby individuals had mistaken conceptions of memory problems and self-worth challenged.<br><br>10 weeks	14	Non-active control	12	UCLA loneliness scale<br><br>Global cognitive score;	Improvement in cognitive scores from pre to post intervention. Trend towards reduction in loneliness. No significant difference compared to control.	y	y	n	y	n
Cohen-Mansfield et al	Volunteers in the community	SMC MMSE >24; Hebrew language; Age>65 years	Health promotion- topics included health behaviours, dementia, cognitive activities, relationships, depression and coping, home and travel safety,	15	Non-active control	12	UCLA loneliness scale<br><br>Global cognitive score;	Improvement in cognitive scores from pre to post intervention. Trend towards reduction in loneliness. No significant difference compared to control.	y	y	n	y	n

			recreation and leisure, medications and health care providers, physical activity and lifelong learning.<br><br>10 weeks										
Hoogenhaut et al	Volunteers in the community	SCD; women; Age 60-75 years	Psychoeducation- information delivered on subjective cognitive difficulties, ageing and internal/external strategies.<br><br>Eight 1.5 hour sessions delivered over 4 weeks.	30	Waiting list control	30	MMI<br><br>Psychological well-being quotient (CES-D, NCQ, SF-36);<br><br>Memory quotient based on VVLT; Executive Functioning and Speed Quotient- (CST, SCWT, LDST)	Participants in the experimental group reported fewer emotional reactions towards cognitive functioning post-intervention.<br><br>No significant differences between groups for psychological well-being, memory or executive function.	y	y	y	y	N

Kwok et al	Volunteers from community centres	SMC; Age >60 years; no physical or psychiatric co-morbidity associated with cognitive impairment; >22 on MMSE	Active mind training programme- education on memory decline and dementia together with cognitive training.<br><br>1 hour, once a week for 8 weeks	86	Active control- usual group activities provided by community centre such as daily discussion about the news, outings and monthly gatherings.	90	Total adjusted CDRS;<br>CDRS scores for attention, Initiation, construction Conceptualisation, SF12-Mental	Significant improvement in mental health subscore on SF 12 questionnaire.<br><br>Significant improvement in overall global cognition with conceptualisation being most marked.	y	y	n	y	Y
Metternich et al	Memory Clinic	Meet criteria for diagnosis of FMD; Age <69; IQ>80;	Psychoeducation, cognitive restructuring, stress management, relaxation and mindfulness techniques.	18	Waiting list control	18	SCL-90 R GSI; PSQ<br><br>MIA	Significant improvement in MSE at 3 month follow up post intervention. No significant difference immediately after the intervention.	y	y	y	y	y
Oh et al	Volunteers in the community	SMC; Smartphone owner; K-MMSE >23	SMART – a smartphone application developed to improve the user's attention and working memory. Tasks have three difficulty levels.	18	Waiting list control	16	MMQ<br><br>CES-D; STAI-S<br><br>MDS	Significant improvement in working memory quotient and auditory-working memory.	y	y	n	y	n

			15-20 minutes daily for 5 days a week for 8 weeks										
Oh et al	Volunteers in the community	SMC; Smartphone owner; K-MMSE >23	Fitbrains-smartphone application providing cognitive training. Three tasks within each session with thirty sessions in total. Recommendation to complete one session daily. Each task was completed twice to increase training time.<br><br>Unclear over what duration training was undertaken but at least 30 days over an 8 week period.	19	Waiting list control	16	MMQ<br><br>CES-D; STAI-S<br><br>MDS	Significant improvement in contentment on MMQ	y	y	n	y	n
Pereira-Morales et al	Volunteers from community centres	MMSE>21; no diagnosis of MCI/ dementia;	Integrated psycho-stimulation programme (IPP):	17	Non-active control-Information brochure on how to reduce risks of	11	SMCQ<br><br>STAI	Significant improvement in metamemory and anxiety in IPP	-	n	-	y	n

		intact ADLs; no significant co-morbid psychiatric or neurological condition.	Cognitive training, progressive relaxation exercises, meta-memory activities and group discussions to engender socialisation..<br><br>90 mins, 4 times a week for 8 weeks		developing MCI/ dementia		Grober and Buschke short/long term memory; Stroop; WAIS	group compared to baseline.					
Pereira-Morales et al	Volunteers from community centres	MMSE>21; no diagnosis of MCI/ dementia; intact ADLs; no significant co-morbid psychiatric or neurological condition.	Computerised cognitive training (CCT).<br>60 mins, 4 times a week for 8 weeks.	12	Non-active control- Information brochure on how to reduce risks of developing MCI/ dementia	11	SMCQ<br><br>STAI<br><br>Grober and Buschke short/long term memory; Stroop; WAIS	Significant improvement in cognition as compared to baseline.	-	n	-	y	n
*Pike et al	Volunteers from community centres	SMC; Age>60; community dwelling; independent ADLs,; English language	Face-name memory training in an office based setting. Either allocated to receive training based on semantic association (SA)	22 (SA)<br><br>25 (SR)	No training	21<br><br>21	Cued/uncued recall.<br><br>MMQ-contentment MCI-memory control beliefs	Spaced retrieval improved recall for cued and uncued names, whilst semantic association improved only recall for cued names. Neither	Y	n	n	n	n

			or spaced retrieval (SR).<br><br>One off 1.5 hour session followed by 2 booster phone calls.					were statistically significant.<br><br>No improvement with either intervention in naturalistic setting when assessed two months later.					
Scogin et al	Volunteers in the community.	SMD; Age > 60;	Self-administered cognitive training from a manual.<br><br>Sixteen one hour study sessions.<br><br>Once weekly phone calls from first author to ensure compliance	20	Waiting list control	27	Memory recall tasks; Benton Visual Retention Test.<br><br>Metamemory Questionnaire.<br><br>Zhung depression scale.	No significant difference in composite measure of metacognition or objective cognitive performance or in depression scores<br><br>No differences in memory complaint scores or depression scores.	n	n	n	y	n
Small et al	Volunteers in the community.	SMI; Age 35-69; absence of severe psychiatric or neurological illness; absence of MCI; not	14-day Healthy lifestyle programme based on mental and physical exercises, healthy diet, and stress reduction techniques.	8	Non-active control.<br><br>Continued lifestyle as usual.<br><br>2 weeks	9	Buschke Fuld Selective Reminding Test; COWAT<br><br>MFQ	Verbal fluency improved significantly in intervention group but no significant difference between groups.	n	n	n	y	n

		taking psychotropic medication	2 weeks					No statistically significant differences between groups.					
Smart et al	Volunteers in the community	SCC; Age 65-85; English language; no significant neurological disease; MMSE ≥25;tolerate MRI;	Mindfulness based therapy<br><br>Eight 2 hour long, once weekly group sessions	8	Active control-psychoeducation<br><br>Five 2 hour long, once weekly group sessions	6	Cognitive complaints index – composite score made from cognitive subscales of GDS, MCQ and MIA<br><br>FFMQ-A	P3 ERP amplitude significantly increased.<br><br>No significant Improvements in self-reported subjective memory complaints, mindful attention measures.	-	y	-	y	n
Tsai et al	Volunteers from community setting.	SMC; Age >55; CDR score 0; Independent ADLs; Chinese language	Cognitive training which included strategies and skills to improve cognitive performance. Also taught meditation and some psychoeducation.<br><br>2 hours, twice weekly for 5 weeks.	14	Active control-cognitive stimulation Which included problem solving exercises and quizzes.<br><br>1.5 hours, once weekly for 8 weeks.	11	ADAS-Cog; MMSE; SRT Clock Drawing Test	Statistically significant improvements in MMSE and ADAS-Cog in both groups at the end of the intervention and 6 months follow up but no significant difference between groups.	n	n	n	y	n

Valentijn et al	Volunteers from community setting.	SMC; Age>55; Dutch language; >24 on MMSE; mobile,	Collective memory training including internal and external memory strategies as well as psychoeducation.<br><br>2 hour weekly session for 8 weeks	53	Waiting list control	43	VVLT; Short story test; CFQ<br><br>MIA	Significant improvement in recall.<br><br>Significant improvement on MIA change and anxiety subscale score.	y	n	y	n	n
Valentijn et al	Volunteers in the community	SMC; Age >55; Dutch language; MMSE >24; mobile	Individual training – subjects given "Memory support" book with the content described in the same order as above. 8-week time period (called 4 weeks through to ensure compliance with the training)	43	Waiting list control	43	VVLT; Short story test; CFQ<br><br>MIA	Significant improvement in recall.<br><br>Significant improvement in MIA anxiety scores.	y	n	y	n	n
*Van Hooren et al	Volunteers in the community.	SCC; Age>55; MMSE≥24; Dutch language; travel independently; absence of	Goal management training-structured and interactive intervention to teach individuals	38	Waiting list control	31	SCWT<br><br>CFQ<br><br>SCL-90	Significant reduction in anxiety scores.<br><br>Significant reduction in annoyance towards cognitive failures.	y	n	y	n	n

		psychiatric or neurological co-morbidity.	a strategy to improve planning activities and to structure intentions. Additionally, there was a psychoeducation component about cognitive ageing.<br><br>12 sessions each 1.5 hours long delivered over 6 weeks (1 individual and 11 group sessions)					Significant reduction in number of executive failures reported.					
*Youn et al	Memory clinic	SMC; Age > 55; excluded if serious psychiatric or medical co-morbidity	Cognitive training (focus on metamemory)<br><br>10 week programme, of 90 min 1x weekly sessions	20	Waiting list control	20	SRFT; Elderly verbal learning test; DST; VST; Phonemic fluency test; Categorical fluency test<br><br>SMCQ<br><br>GDS	Significant improvements on short and long term recall, SRFT recognition, VST (forward), categorical fluency.	y	Y	n	y	n

Zhu et al	Volunteers in the community	SCC; Age ≥50 years, not on psychotropic medication, , no severe psychiatric or medical conditions	Brain Power Advanced supplement.<br><br>2 capsules with meals for 12 weeks.	47	Non-active control- placebo tablets.<br><br>2 capsules with meals for 12 weeks.	51	Visual, auditory and short-term memory<br><br>Subjective memory loss and subjective attentional deficits.	No significant difference between in subjective memory loss or subjective attentional deficit scores.	Y	y	n	y	n

SMC, Subjective memory complaints; MDRS, Mattis Dementia Rating Scale; RAVLT, Rey Auditory Verbal Learning Test; EFT, Eriksen Flanker Test; DSST, Digit Symbol Substitution Test; TMT-A, Trail Making Test A; TMT-B, Trail Making Test B; UFOV, Useful field of view; TSST, Trier Social Stress Test; SMI, subjective memory impairment; PRMQ, Prospective and Retrospective Memory Questionnaire; EMCT, Expanded Mental Control Test; SCD, Subjective Cognitive Decline; MMI, Maastrict Metacognition Inventory; CES-D, Centre of Epidemiologic Studies Depression scale; NCQ, Neurovegetative Complaints Questionnaire; SF-36, Short-Form Health Survey; CST, Concept Shifting Test; SCWT, Stroop Color-Word Test; LDST, Letter Digit Substitution; VVLT, Visual Verbal Learning Test; SF12, 12-Item Short Form Health Survey; CDRS, Chinese version of Mattis Dementia Rating Scale; SCL-90 R GSI, Symptom Checklist 90-R; MIA, Metamemory in Adulthood Questionnaire; PSQ, Perceived Stress Questionnaire; FMD, Functional Memory Disorder; WAIS, Wechsler Adult Intelligence Scale; STAI, State-Trait Anxiety Inventory; SMCQ, Subjective Memory Complaints Questionnaire; MMQ, Multifactorial Memory Questionnaire; MDS, Memory Diagnostic System; MCI, Mild Cognitive Impairment; SRT, Selective Reminding Test; MMSE, Mini-Mental State Examination; CDR, Clinical Dementia Rating; ADAS-cog, Alzheimer's disease Assessment Scale-Cognition; CFQ, Cognitive Failure Questionnaire; SRFT, Simple-Rey figure test; DST, Digit Span Test; VST, Visual Span Test; MCQ, Memory Complaints Questionnaire; FFMQ-A, five facet mindfulness questionnaire- "mindful attention" subscales; COWAT, Controlled oral word association test; MFQ, Memory Function Questionnaire; SMI, Subjective Memory impairment.
***Not included in meta-analysis**
