# Supplementary material for: Interventions for subjective cognitive decline: systematic review and meta-analysis
Source: BMJ Open. 2018 Jul 19;8(7):e021610. doi: 10.1136/bmjopen-2018-021610 (PMC6059327; doi:10.1136/bmjopen-2018-021610)
Supplement: Supplementary file 1 [file bmjopen-2018-021610supp001.pdf]

Supplementary Appendix 1

Search Strategy (used in PubMed)

(“intervention” OR “treatment” OR “rehabilitation” OR “cognitive therapy” OR “cognitive training” OR “memory training” OR “computerised” OR “exercise” OR “cognitive behavioural” OR “cognitive behavioural” OR “metacognit*” OR “memory self- efficacy” OR “metamemory” OR “goal management” OR “expectancy change” OR “psychological” OR “group” OR “cognitive” OR “mindfulness” OR “Mediterranean diet” OR “mindfulness” OR “acupuncture” OR “meditation” OR “lifestyle” OR “diet” OR “gingko” OR “complementary” OR “vitamin*” OR “supplement*” OR “acetylcholinesterase inhibitor” OR “donepezil” OR “memantine” OR “galantamine” OR “rivastigmine”)

AND (“randomised controlled trial” OR “controlled trial” OR “intervention trial” OR “intervention study” OR “experimental study” OR “crossover trial” OR “RCT” OR “parallel”)

AND (“subjective cognitive decline” OR “subjective memory complain* OR “subjective cognitive concern” OR “subjective cognitive complain* OR “SCD” OR “SMC” OR “SCC” OR “preclinical dementia” OR “preclinical Alzheimer” OR “functional” OR “benign” OR “healthy”)

Statistical methods

Effect size calculation

Effect sizes were calculated using RevMan software. Standardised mean differences were calculated using Hedges’ adjusted g.

Pre-intervention standard deviations were used as these are most likely to be comparable across studies and therefore provide the most accurate estimate of effect size.[53]

The Hedges’ adjusted g formula[26] used in RevMan is as follows:

$g= [M_{post\ intervention} – M_{post\ control}/SD_{pre\text{-}pooled}]*[1- 3/(4N-9)]$

Where $N= n_{intervention\ group} + n_{control\ group}$

and

$SD_{pre\text{-}pooled} = \surd\ [((n_{intervention\text{-}1})\ SD_{pre\ intervention}^2 + (n_{control\text{-}1})\ SD_{pre\ control}^2)/\ N-2]$

Meta-analyses

Meta-analyses were performed using RevMan software. A random effects method as described by DeSirmonian and laird[54] was used. This adjusts standard errors of the effect sizes in each study to account for the heterogeneity for intervention effects observed between different studies.

The pooled effect size of each meta-analysis was calculated by attributing a weight to the average effect size in each study according to sample size. The z statistic was used to evaluate whether the pooled effect size was significantly different to no effect.

Heterogeneity was quantified using the I^s statistic.

Composite measure calculation

Composite scores were calculated where a study reported multiple outcomes falling within a particular outcome domain (e.g. objective cognitive performance). This approach was pragmatic in allowing one score to represent each intervention in the meta-analysis regardless of the number of outcomes reported.[27] The variance of the sum of variables was calculated as described below.

For example, if a study reported two relevant outcomes, expressed as two effect sizes (y_1 and y_2). The overall mean effect size for the composite measure will be:

$\bar{y}$= ½ (y_1 + y_2)

The variance of the composite effect size is calculated as follows:

$V_{\bar{y}}$= ¼ (V_{Y1} + V_{y2} + 2r*√V_{Y1}*√ V_{y2}),

where r is the correlation coefficient describing to what extent y_1 and y_2 co-vary.

If the correlation is set at 0, the outcomes are essentially treated as independent of each other and if the correlation is set at 1, the variance is an average of each outcome's variance. The former will lead to an underestimation of the variance and overestimation of precision while the latter will have the opposite effect. Consequently, in the absence of existing literature to identify a suitable correlation, we reported composite effect sizes calculated using a correlation of 0.5.

Supplementary appendix 2

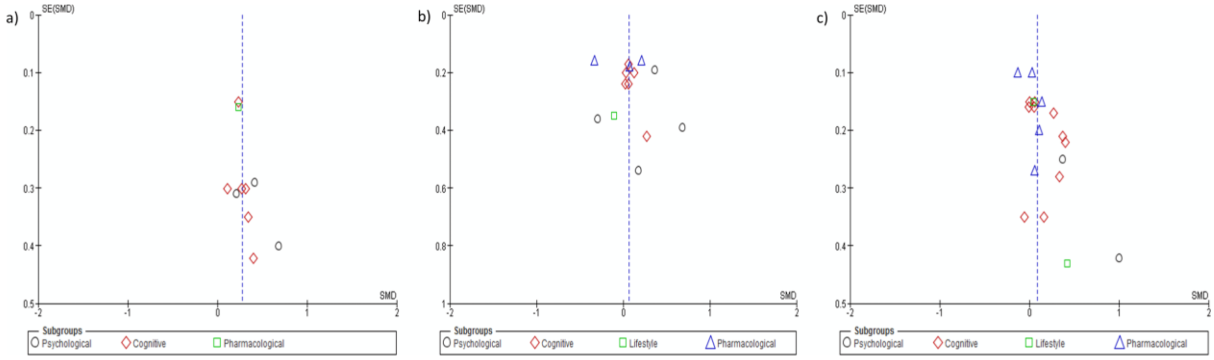

Figure DS1. Funnel plot as indicator of publication bias. All studies for a) psychological well-being outcomes; b) metacognitive ability outcomes and c) objective cognitive performance outcomes.

Author	Intervention	Standardised mean difference (95% CI)
Cohen-Mansfield et al[36]	Group cognitive training	0.16[-0.53, 0.85]
Kwok et al[37]	Cognitive training program	0.06 [-0.23, 0.35]
Tsai et al[40]	Cognitive training	-0.06 [-0.75, 0.63]
**Overall**	**Global cognitive performance**	0.06 [-0.19, 0.31]

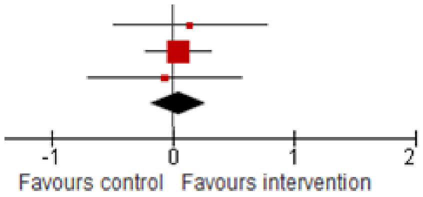

Heterogeneity: $Tau^2 = 0.00$; $Chi^2 = 0.20$, df = 2 (P = 0.91); $I^2 = 0\%$
Test for overall effect: Z = 0.45 (P = 0.66)

Fig. DS2 Forest plot comparing cognitive interventions vs control (all types). Outcome: Objective global cognitive performance (at the end of intervention).

Supplementary References

26. Hedges LV, Olkin I. Statistical methods for meta-analysis. New York, Academic Press 1985.

27. Borenstein M, Hedges LV, Higgins JPT, et al. Introduction to Meta-Analysis. Chichester, John Wiley & Sons, Ltd 2009.

53. Morris S. Estimating Effect Sizes From Pretest-Posttest-Control Group Designs. Organ. Res. Meth 2008;11(2):364-386.

54. DerSimonian R, Laird N. Meta-analysis in clinical trials. Control Clin Trials 1986; 7(3):177-188
