# Supplementary material for: Interventions for subjective cognitive decline: systematic review and meta-analysis
Source: BMJ Open. 2018 Jul 19;8(7):e021610. doi: 10.1136/bmjopen-2018-021610 (PMC6059327; doi:10.1136/bmjopen-2018-021610)
Supplement: Author's manuscript [file bmjopen-2018-021610.draft_revisions.pdf]

BMJ Open

BMJ Open is committed to open peer review. As part of this commitment we make the peer review history of every article we publish publicly available.

When an article is published we post the peer reviewers' comments and the authors' responses online. We also post the versions of the paper that were used during peer review. These are the versions that the peer review comments apply to.

The versions of the paper that follow are the versions that were submitted during the peer review process. They are not the versions of record or the final published versions. They should not be cited or distributed as the published version of this manuscript.

BMJ Open is an open access journal and the full, final, typeset and author-corrected version of record of the manuscript is available on our site with no access controls, subscription charges or pay-per-view fees (http://bmjopen.bmj.com).

If you have any questions on BMJ Open's open peer review process please email info.bmjopen@bmj.com

BMJ Open

Interventions for Subjective Cognitive Decline: Systematic Review and Meta-analysis

Journal:	*BMJ Open*
Manuscript ID	bmjopen-2018-021610
Article Type:	Research
Date Submitted by the Author:	12-Jan-2018

Complete List of Authors:	Bhome, Rohan; University College London, Division of Psychiatry<br>Berry, Alex; Camden and Islington NHS Foundation Trust<br>Huntley, Jonathan; University College London, Division of Psychiatry<br>Howard, R; University College London Division of Psychiatry,

Keywords:	Subjective cognitive decline, Metacognition, Group interventions, Psychologcial well-being, Systematic review

SCHOLARONE™
Manuscripts

For peer review only

Interventions for Subjective Cognitive Decline: Systematic Review and Meta-analysis

Title Page

Title: Interventions for Subjective Cognitive Decline: Systematic Review and Meta-analysis

First and corresponding author: Rohan Bhome, Division of Psychiatry, University College London, London, UK. rohan.bhome@ucl.ac.uk

2nd author: Alex J Berry, Camden and Islington Foundation Trust, London, UK

3rd author: Jonathan D Huntley, Division of Psychiatry, University College London, London, UK

Last author: Robert J Howard, Division of Psychiatry, University College London, London, UK

Word count: 4003 words

PROSPERO registration number: CRD42017079391

For peer review only

For peer review only - http://bmjopen.bmj.com/site/about/guidelines.xhtml

ABSTRACT

Objectives: This review provides a broad overview of the effectiveness of interventions for Subjective Cognitive Decline (SCD) in improving psychological well-being, metacognition and objective cognitive performance.

Method: Online literature databases were searched up to August 2017 and reference lists of relevant articles were manually scanned for potential randomised control trials (RCTs) of interest. Data was extracted and study quality rated independently by three authors. Interventions were categorised as psychological, cognitive, lifestyle or pharmacological. Outcomes of interest included psychological well-being, metacognitive ability and objective cognitive performance. Random effects meta-analyses were undertaken where three or more studies investigated similar interventions and reported comparable outcomes. Where appropriate, separate analyses were carried out for studies using 'active' and 'non-active' controls.

Results: Twenty studies met inclusion criteria and 16 had sufficient data for inclusion in the meta-analyses. Of these, only seven were rated as being high quality. Group psychological interventions significantly improved psychological well-being (g=0.40, 95% CI 0.03-0.76; P=0.03) but the improvement they conferred on metacognitive ability was not statistically significant (g=0.26, 95% CI -0.22-0.73; P=0.28). Overall, cognitive training interventions led to a small, statistically significant improvement in objective cognitive performance (g=0.13, CI 0.01-0.25; P=0.03). However, the pooled effect sizes of studies using active control groups (g=0.02 CI -0.19-0.22; P=0.85) or reporting global cognitive measures (g=0.06, CI=-0.19-0.31; P=0.66) were equivocal. Lifestyle and pharmacological interventions were ineffective.

Conclusions: There is a lack of high quality evidence in this field. Group psychological interventions improve psychological well-being and are likely to improve metacognition. A large, high quality study is indicated to investigate this further. There is no evidence to suggest that cognitive interventions improve global cognitive performance and the clinical utility of small improvements in specific cognitive domains is questionable. There is a lack of evidence considering lifestyle interventions and unconvincing and poor quality evidence for pharmacological interventions.

Strengths and Limitations of this study

This is a comprehensive systematic review, including meta-analysis, of the effectiveness of psychological, cognitive, lifestyle and pharmacological interventions in Subjective Cognitive Decline (SCD).

Rather than solely focussing on cognitive outcome measures, this systematic review also evaluates the effectiveness of the interventions on psychological well-being and metacognition as the former is a more naturalistic outcome while the latter is an important component of SCD.

This systematic review highlights the heterogeneity of previous intervention studies in this field ranging from the exact nature of the intervention, the use of adequate placebo control groups and the outcomes measured.

The studies included in the systematic review are generally of a low quality and this makes it difficult to draw firm conclusions.

For peer review only - http://bmjopen.bmj.com/site/about/guidelines.xhtml

INTRODUCTION

Subjective cognitive decline (SCD) describes self-reported acquired difficulties with cognitive functioning, in the presence of normal performance on objective measures of cognition and no impairment of daily functioning.[1] SCD has attracted alternative names, including subjective memory impairment (SMI) and subjective memory decline (SMD). A significant proportion of patients with SCD are diagnosed as having functional memory disorder (FMD)[2] which describes reversible memory complaints arising secondary to emotional and psychosocial stressors.[3]

The reported prevalence of SCD is variable ranging from 18%-55% of memory clinic attendees[4-7] and between a quarter and half of all people aged over 65 living in the community are affected.[8]

SCD may represent pre-clinical Alzheimer's disease (AD), with sufferers being twice as likely to develop dementia.[9] Patients with SCD have increased amyloid deposition in their brains[10,11] as well as brain changes indicative of AD in imaging studies.[12,13] A recent observational study, however, found only 14% of patients with SCD progressed to mild cognitive impairment (MCI) or AD over a six year follow-up[14] whilst a systematic review[15] highlighted that SCD symptoms frequently remit. These findings reflect the aetiological heterogeneity of SCD which encompasses functional psychiatric illness, personality traits, physical illness, psychosocial stress and the effects of alcohol and drug use as well as early neurodegenerative disease.[1]

Effective interventions for patients with SCD are necessary. For those with pre-clinical AD the significant social, personal and economic impact of dementia[16] make treatments that could slow or even prevent progression to AD particularly important. Preserved cognitive functioning[17] at this earlier pre-clinical stage enables better engagement with interventions. Furthermore, increased cognitive flexibility and malleability could optimise the functioning of compensatory cognitive pathways and offset subtle subclinical deficiencies in others[18] in response to targeted interventions.

Regardless of aetiology, the symptoms experienced by patients with SCD are distressing. Self-perception of cognitive difficulties can lead to anger, increased stress and fear of dementia.[19] Such a state of heightened psychological distress predisposes to further cognitive difficulties and a reinforcing cycle may ensue.[19] Aside from those with an obvious treatable cause of their SCD, such as a co-morbid mood disorder, there is no evidence based consensus on how to best treat this patient group.

Metacognition, an individual's insight into their own cognitive functioning,[20] is relevant because, by definition, SCD involves an individual's underestimation of their cognitive ability. A cross sectional study[21] found that patients who had what a condition analogous to SCD without pre-clinical AD had significantly poorer metacognitive abilities than controls.

Three systematic reviews evaluating interventions for SCD have been published.[19,22,23] Metternich and colleagues'[19] found that 'expectancy change', which included interventions such as cognitive restructuring or psychoeducation to change beliefs about cognition, was significantly more effective at improving metacognition than a non-active control intervention. A limitation of this systematic review was that nine of the 14 studies included involved participants who were healthy volunteers and did not necessarily have SCD. Cavenelli[22] reviewed six studies of cognitive interventions for SCD and found improvements in objective cognitive performance, often limited to the cognitive domain being trained. The most recent systematic review[23] on this topic, perhaps benefitting from being the

For peer review only - http://bmjopen.bmj.com/site/about/guidelines.xhtml

first since the conceptualisation of the term SCD,[1] used a more rigorous inclusion criteria and found a small effect size for all non-pharmacological interventions (NPI).

Purpose of current review

There is no consensus on the best treatment for SCD. We aimed to conduct a systematic review of all types of interventions (cognitive, lifestyle, psychological and pharmacological) and to evaluate their effects on psychological well-being, metacognition and objective cognitive performance.

Since the last systematic review on NPI for this patient group, two further randomised control trials have been published.[24, 25] While cognitive performance outcomes are of value in measuring the efficacy of cognitive training interventions, the clinical utility of small improvements in these domains is questionable.[26] Therefore a focus of this review was to consider the effects on psychological well-being and metacognition because the former represents a more naturalistic outcome measure and deficits in the latter may underpin SCD. We also included pharmacological interventions including those from complementary medicine in this review because their role has not been systematically evaluated previously.

For peer review only

METHODS

Search Strategy and selection criteria

During August 2017, we searched PubMed, Web of Science, Cochrane Systematic Reviews Database, PsycINFO and CIANHL. The key words used were: “memory”, “cognitive” or “forgetfulness” AND “subjective”, “functional”, “benign”, “self-reported”, “complaints”, “complainer” or “healthy” AND “training”, “intervention”, “treatment”, “therapy” or “medicine”. We did not apply limits for language or date of publication. Furthermore, reference lists of included studies were manually scanned. If relevant outcome data were not included in the published article, authors were contacted and data requested to enable inclusion of the study in the meta-analyses.

Inclusion and Exclusion Criteria

We included studies where participants, either all or a separately analysed subgroup, had SCD. Many studies predate the conceptualisation of SCD and used alternative nomenclature. Studies including patients with mild cognitive impairment (MCI) or dementia were excluded as were those including patients with significant psychiatric or physical co-morbidities. There was no age restriction given that neurodegeneration is not the aetiology of most cases of SCD. Only RCTs were included. The intervention delivered needed to fall within the domains of psychological, cognitive, lifestyle or pharmacological. Outcomes of interest included psychological well-being, metacognition and objective cognitive performance.

Assessment of trial Quality

In order to assess the risk of bias, three authors (RB, AB and JH) independently rated study validity using criteria that were based on the Critical Appraisal Skills Programme (CASP; www.hello.nhs.uk/documents/CAT6-Randomised_Controlled_Trials.pdf) and have been used previously.[27,28]

Five criteria assessing quality and bias were assessed and a score out of five for each study was produced (see supplementary appendix 1 for further details on quality assessment).

Data extraction

The studies’ demographic details, participant characteristics, main findings, limitations and results in the form of means and standard deviation for each outcome measure were independently extracted by three authors (RB, AB and JH).

Statistical analysis

We compared intervention and control groups post-intervention using RevMan software. The programme uses Hedges adjusted g to calculate a standardised mean difference (SMD) which is adjusted for small sample bias. Meta-analyses were performed where three or more studies investigated a comparable intervention and outcome using a random effects model. Heterogeneity was quantified using the I^2 statistic.

Where a study reported multiple outcome measures for one outcome domain (eg within psychological well-being) we calculated a composite measure. This overcame the problem of more

For peer review only - http://bmjopen.bmj.com/site/about/guidelines.xhtml

weight being attributed to studies with more outcomes.[29] Further details of statistical methods are found in the supplementary material (see supplementary data file 1).

For peer review only

For peer review only - http://bmjopen.bmj.com/site/about/guidelines.xhtml

RESULTS

Description of studies

Sixty-three potentially relevant articles were retrieved through our initial search. Of these, 20 studies met inclusion criteria, however only 16 studies had sufficient outcome data to allow calculation of effect sizes for inclusion in meta-analyses (Figure 1). The characteristics and validity of included studies are described in the online supplementary material (supplementary data file 2: Table DS1).

Of the twenty included studies, five were classified as psychological interventions; four as group psychological treatment[25,30-32] and one as mindfulness training.[33] Eleven studies were classified as cognitive or memory training,[24,25,34-42] two as lifestyle[35,43] and four as pharmacological[44-47] interventions. Three studies[24,32,46] examined two differing interventions from the same category while two studies investigated interventions from different categories.[25,35]

Participants were community dwelling in all of the studies although the source of recruitment varied; memory clinics in two studies,[31,42] community centres in three studies[25,37,38] and the remainder through more widespread advertisement.

Of the RCTs included in the meta-analysis, twelve had a non-active control[24,25,30,31,36,39,41,43-47] while four had an active control.[33,35,37,40] The studies included a range of outcomes and only five RCTs[30,31,35,37,44] clearly stated their primary outcome a priori. Fifteen RCTs[24,25,30,32,35-37,39-41,43-47] included in the meta-analysis included objective cognitive performance amongst their outcomes but only three of these used an assessment of global cognitive function.[36,37,40] Ten studies[24,25,31-34,38,39,41,43,46,47] reported metacognitive outcomes, of which three provided a global measure of metacognition.[25,31,41] Seven studies[24,25,30,32,36,39,44] reported psychological well-being outcomes. All of the RCTs reported outcome data within two weeks of the intervention ending and four studies[31,39-41] collected further follow-up data up ranging from one to six months post-intervention.

As stipulated *a priori*, meta-analyses were performed where three or more studies included comparable interventions and reported comparable outcomes. This meant that the effectiveness of psychological interventions on psychological well-being and metacognition as well as cognitive interventions on psychological well-being, metacognition and objective cognitive performance were meta-analysed.

Quality of studies

According to criteria described in the methods section, seven studies[30,31,35,41,43,44,47] included in the meta-analyses were rated as being of high quality. An a priori power calculation was only carried out in two studies[31,35] while the analysis of incomplete outcome data was adequately addressed in only eight studies.[30,31,35,41,43,44,47] Thirteen[24,30-32,35-37,39,41-47] out of 16 studies met criteria for adequate randomisation of participants and the same number of studies[24,30,31,33,35,36,39,41-47] ensured adequate blinding of participants and researchers.

Group psychological interventions (four studies)

Four small studies[25,30-32] investigated psychological interventions. Three[30-32] were assessed to be high quality. Their main focus included psychoeducation about cognitive ageing and the link between cognitive difficulties and stress or anxiety together with goal management, strategies to improve metacognition and relaxation training. The intervention was delivered either completely[30-32] or partially[25] in group settings. One RCT,[32] which showed a reduction in anxiety symptoms in the intervention group compared to the control group, could not be included in meta-analyses due to

For peer review only - http://bmjopen.bmj.com/site/about/guidelines.xhtml

insufficient baseline data. The meta-analyses for the three remaining studies showed a significant improvement in psychological well-being post-intervention (g=0.40, 95% CI 0.03-0.76, Z=2.12, P=0.03, see figure 2). Considering metacognition, in Van Hooren's RCT,[32] participants who received the intervention managed cognitive failures better (F (1,62)= 4.32, p=0.04) than those in the control group. In combination, the three remaining studies looking at group psychological interventions found a positive effect on metacognition but this was not statistically significant (g=0.26, 95% CI -0.22-0.73, Z= 1.07, P=0.28, see figure 3). Interestingly, Metternich and co-workers[31] found that their group based therapy did not improve memory self-efficacy (MSE) immediately post-intervention but there was a significant improvement in MSE at three months follow up (P=0.04).

Three of the studies[25,30,32] measured objective cognitive performance. Hoogenhout et al[30] did not find any significant differences in objective measures of memory (U=229, p=0.161) or executive functioning and speed (U=274, p=0.616). Similarly there was no effect on executive function, the only cognitive domain evaluated by Van Hooren et al.[32] In Pereira-Morales' study[25] the Integrated Psychostimulation Program (IPP), which contains cognitive training, conferred a significant improvement in a composite measure of objective cognitive performance (see figure 4).

Mindfulness (One study)

Smart et al[33] conducted a pilot RCT to assess the feasibility of mindfulness in SCD. The number of participants with SCD was low (n=15). A key limitation of this study was that the control group received psychoeducation which in itself has evidence in improving psychological well-being and metacognition in this patient group. Both groups demonstrated an improvement in MSE and a reduction in cognitive complaints with no significant difference between them.

Lifestyle interventions (two studies)

A high quality, adequately powered, factorial design RCT[35] showed no benefit of aerobic exercise on objective cognitive performance compared to an active control (gentle exercise that did not increase heart rate) (g=0.04, CI -0.25-0.33, see figure 4). However, there was significant improvement in both groups.

Small's[43] small but high quality RCT found that an intervention to promote healthy lifestyle strategies did not improve metacognition (g=-0.11, CI -0.80-0.58, see figure 3). There was an improvement, albeit not reaching statistical significance, in a composite measure of verbal learning and memory and verbal fluency (g=0.42, CI -0.42-1.26, see figure 4). The small sample size and lack of monitoring of the compliance of participants prevents any firm conclusions being drawn. Of note, the study included patients aged 35-69 as there were no age restrictions.

Cognitive training interventions (11 studies)

Thirteen cognitive or memory training interventions across 11 RCTs[24,25,34-42] were evaluated. Three studies[34,38,42] provided insufficient data to be included in meta-analyses. Only two studies[35,41] were rated as high quality. For objective cognitive performance, significant pooled effect sizes were found (g=0.13, CI 0.01-0.25, Z=2.13, P=0.03 see figure 4) with no heterogeneity (I^2=0%) between studies. Three studies[35,37,40] had active control groups in which participants received forms of cognitive stimulation and when analysed separately, their pooled effect size was equivocal (g=0.02 CI -0.19-0.22, Z=0.19, P=0.85, see figure 5). Conversely, for the remaining studies which used non-active controls, the pooled effect size was significant (g=0.18, CI 0.04-0.33, Z=2.48, P=0.01, see figure 5).

For peer review only - http://bmjopen.bmj.com/site/about/guidelines.xhtml

Three studies[36,37,40] used global cognitive performance outcome measures and when meta-analysed there was no discernible improvement post intervention (g=0.06, CI=-0.19-0.31, Z=0.45, P=0.66, see supplementary appendix 2: Figure DS1).

Five studies[24,25,36,37,39] reported psychological well-being. Each of the studies demonstrated an improvement in psychological well-being but individually none were statistically significant. However, when meta-analysed there was a small beneficial effect on psychological well-being (g=0.25, CI= 0.05-0.46, Z=2.40, P=0.02, see figure 2) with no heterogeneity (I^2=0). Three studies[24,39,41] reported metacognitive outcomes. A total of five different cognitive or memory training interventions each showed a very small, insignificant improvement in metacognition which resulted in a non-significant pooled effect size (g=0.06, CI -0.12-0.24, Z=0.66, P=0.51, see figure 3).

Youn et al[42] used a memory training programme which incorporated strategies to improve metacognition. This small, low quality RCT found no significant effect on metacognition or psychological well-being but there were significant improvements in various domains of memory functioning.

Pharmacological interventions (4 studies)

Two studies investigated the effectiveness of Gingko biloba. Brautigam's low quality but relatively large trial[46] included two different dosing regimens, namely high and low dose Gingko extract for 24 weeks. They led to conflicting results with high dose Gingko causing a statistically significant worsening in metacognition while low dose Gingko produced the opposite effect although statistically insignificant.

In Beck and colleagues' trial,[44] a 240mg preparation of Gingko Biloba was administered to the intervention group daily for eight weeks. They found improvements in both cognition (g= 0.14, CI -0.15-0.43, see figure 4) and psychological well-being (g=0.23, CI -0.08-0.54, see figure 3) but neither was statistically significant.

One small, low quality trial by Boespflug et al[45] investigated the effects of 2.4g fish oil daily on working memory. This was a secondary outcome in this study and the results were non-significant (g=0.06, CI -0.47-0.59, see figure 4).

Zhu and colleagues[47] evaluated the effectiveness of BrainPower Advanced, a dietary supplement, taken twice daily for 12 weeks. The trial was of a poor quality with marked baseline differences in severity of cognitive complaints between groups. Analysing the primary data, there was no significant difference in metacognitive ability (g=0.07, CI -0.35-0.31, see figure 3) or objective cognitive performance (g=0.11, CI -0.28-0.50, see figure 4) between the groups.

For peer review only - http://bmjopen.bmj.com/site/about/guidelines.xhtml

DISCUSSION

Main Findings

The most obvious finding from our review is the lack of good quality evidence in this field. However, given that that this is an emerging field, we attempted to be as inclusive as possible and included low quality studies in our meta-analyses provided they did not have an inadequate or unclear rating in all five areas of bias that we assessed (see supplementary data file 1).

There was a statistically significant improvement in psychological well-being for interventions which incorporated psychoeducation and were delivered in group settings, although the effect size was only moderate (g=0.40).[48] The studies, two of which were of high quality, are limited by small sample sizes and one of the interventions[25] included cognitive training in addition to a psychological approach. Overall, however, this is a promising, cost-effective intervention that can be feasibly implemented into clinical practice. Furthermore, psychological well-being is an important and clinically relevant outcome in this patient group.[20]

We found that cognitive interventions conferred a small but statistically significant effect (g=0.13) on cognitive outcomes which is in keeping with Smart's earlier review.[23] However, the clinical relevance of such a small improvement is easy to question. The apparent clinical utility of cognitive interventions in SCD is further undermined by our finding that meta-analysis of the three studies which used global cognitive performance outcome measures found no overall benefit. Our use of composite measures provided us with a single effect size for cognitive performance in each study but we are mindful that this merely reflects performance across the cognitive domains assessed and is not a surrogate for global cognition. Smart[23] suggested that the small improvements in cognition are important because they demonstrate that cognitive enhancement and compensation is possible in this group of patients. While this may be of relevance when considering the small but significant minority of persons in whom SCD represents pre-clinical AD, this finding has little bearing on the remaining majority who, by definition, have normal objective cognitive performance. Future research on patients with SCD would benefit by discerning between those with and without pre-clinical AD.

We found that cognitive training conferred a small but significant benefit on psychological well-being. Of the five cognitive studies reporting psychological well-being measures, only the most highly powered study by Kwok[35] had stipulated a primary outcome and this was not for psychological well-being. The effects of cognitive training on psychological well-being have been poorly researched[49] and there is sometimes an unproven assumption that cognitive training enhances a patient's overall well-being by preserving cognitive function.[50] This would not explain our findings given patients were evaluated within a short time frame from baseline. The larger effect on psychological well-being than on objective cognitive performance measures may reflect a significant psychogenic aetiology in many cases of SCD. Alternatively, SCD may represent a construct in which cognitive deficits are too subtle to be detected by the neuropsychological batteries used. However, it is important to note that the evidence is of a poor quality and this may represent a chance finding.

Metacognitive deficits are a central component of subjective cognitive disorders.[21] Psychoeducation delivered in group settings did not lead to a significant improvement in metacognition immediately after the intervention. This finding was influenced by Metternich and colleagues'[31] study which found less improvement in the intervention group compared to the control (g=-0.30, CI= -1.01-0.41). However, their intervention led to a significant improvement in metacognition when assessed three months post-treatment compared to baseline leading them to postulate that a period of time is

For peer review only - http://bmjopen.bmj.com/site/about/guidelines.xhtml

needed for cognitive restructuring and the implementation of strategies into everyday life. Indeed, an earlier systematic review,[20] which was not restricted to participants with SCD, found that cognitive restructuring along with psychoeducation targeted towards memory functioning was effective at improving subjective memory performance. Our findings suggest that further work is required to explore the use of group psychoeducation in improving metacognition and longer lengths of follow up are indicated. A limitation to the literature investigating metacognitive outcomes is the broad range of subjective measures in use. Recent development of more objective measures of metacognitive performance[20] could improve this.

There is only limited evidence available with regards to lifestyle interventions for SCD. Exercise was evaluated in one, high-quality RCT. The intervention group performed aerobic exercise thrice weekly for an hour for twelve weeks while the control group performed gentle exercise designed not to increase heart rate. Both groups showed significant improvements in objective cognitive performance but there was no difference between the groups. This may suggest that exercise, regardless of intensity, could be beneficial in SCD and further RCTs with a non-active control group are indicated. A healthy lifestyle intervention investigated by Small and colleagues[43] did not confer any benefits on psychological well-being or objective memory performance but was limited by a lack of monitoring of participants.

Five studies have investigated alternative medicines and medicinal supplements in SCD. Overall, the evidence for their efficacy is not encouraging. One high quality study found that 240mg daily of Gingko Biloba for eight weeks led to non-significant improvements in psychological well-being and cognitive performance. Brautigam and colleagues[46] used two alternative doses of Gingko Biloba and their results are conflicting. Higher doses led to significantly worse m compared to control and lower doses led to statistically insignificant improvements. Fish oil[45] was not found to improve objective cognitive performance while a dietary supplement investigated by Zhu et al[47] did not improve either metacognition or objective cognitive performance.

In summary, psychoeducation delivered in group settings appears to be effective at improving psychological well-being, which is a relevant and important outcome, in SCD. However, individually none of the three studies demonstrated statistically significant improvements which was largely due to their small sample sizes. A large RCT is urgently needed to extrapolate our findings for this clinically practical intervention which is highly promising. Interestingly, psychological well-being improved following cognitive training but this was not a primary outcome in any of the five studies. Metacognition, an individual’s insight into their own cognitive functioning, is a potential area of research in SCD because deficits in this area may contribute to the condition, especially the non-preclinical dementia type. Specific interventions to improve metacognitive performance as well as to objectively quantify it are emerging[20] and these may be of relevance in SCD. Cognitive interventions have a modest effect in improving objective cognitive performance and such small improvements are unlikely to be of any clinical significance for the vast majority of patients with SCD who are not at risk for developing dementia. There is limited evidence considering lifestyle interventions in SCD and only unconvincing and poor quality evidence for pharmacological interventions in SCD.

Limitations

SCD has only recently been conceptualised[1] and is a construct which includes a heterogenous group of patients broadly divided into two distinct patient groups; those with and without preclinical dementia. In the absence of widespread testing for implicating genes, neurodegenerative markers and detailed neuroimaging, it will remain difficult to distinguish these groups. All of the studies in our review included both groups of SCD patients and this limits our ability to make inferences about

For peer review only - http://bmjopen.bmj.com/site/about/guidelines.xhtml

targeted interventions for each group. Future work should investigate interventions for each group of patients separately although adequately powering such studies may be challenging.

The poor quality of studies in this review due to a lack of clearly defined primary outcomes and small sample sizes has been highlighted previously and limits the confidence with which we can draw conclusions.

The interventions were heterogenous and no two studies investigated the same intervention. As well as differing formats and structures of interventions which we attempted to group into broad categories, there were differences in lengths of intervention, monitoring of compliance and the motivation of participants which are more difficult to quantify. Often interventions had multiple components and we had to determine which domain was the most prominent. This may mean the most effective component of the intervention was not appropriately captured.

Similarly, the studies included in the meta-analysis contained varied control groups. In at least two[33,40] of the four studies using an active control group, the control group received treatment which may have been effective in its own right thereby masking the possible effectiveness of the intervention. The other studies used non-active control groups but there is likely to have been much variability in what treatment as usual constituted. There is a definite need for well-designed and theoretically based control interventions, especially in a field such as SCD where a gold standard treatment does not exist.

The goal of research into interventions must be for them to be translational into clinical practice. In this sense, it would be important for the participants in a study to represent patients with SCD whose difficulties have reached a threshold to access healthcare. Unfortunately, only one study[31] included in the meta-analyses recruited patients from a healthcare setting whilst the remainder relied on convenience sampling of volunteers with subjective cognitive complaints from the community.

Implications

This review demonstrates the need for higher quality studies in SCD research with a distinction made between SCD in pre-clinical AD and otherwise. The development of a conceptual framework for research in SCD[1] should aid this. This review would support high quality RCTs of a group psychological intervention targeting psychological well-being and metacognition in patients with SCD. Follow-up data at least three months post-intervention should be collected. Metacognition is an important aspect of SCD and more novel interventions to directly improve this along with objective measurement tools are required.

For peer review only

For peer review only - http://bmjopen.bmj.com/site/about/guidelines.xhtml

Contributors

All authors made a substantial contribution to this work. RB, AB, JH and RH all contributed to the conception and design of the review. RB read and screened abstracts and titles of potentially relevant studies. RB, AB and JH read the retained papers and were responsible for extracting data and rating their quality independently. RB drafted the paper with all the authors critically reviewing it and suggesting amendments prior to submission. All the authors had access to all the data in the study and can take responsibility for the integrity of the reported findings.

Funding

This research received no specific grant from any funding body. RB is supported by a NIHR Academic Clinical Fellowship. JH and RH are supported by the NIHR UCLH BRC.

Competing Interests

None declared.

Data sharing statement

Details of excluded papers are available from the first author on request.

For peer review only

For peer review only - http://bmjopen.bmj.com/site/about/guidelines.xhtml

Figure legends

Figure 1. Details of search strategy. RCT, Randomised control trial.

Figure 2. Forest plot comparing interventions (all types) vs control (all types) for patients with subjective cognitive decline. Outcome: Psychological well-being (at the end of intervention).

Figure 3. Forest plot comparing interventions (all types) vs control (all types) for patients with subjective cognitive decline. Outcome: Metacognitive ability (at the end of intervention).

Figure 4. Forest plot comparing interventions (all types) vs control (all types) for patients with subjective cognitive decline. Outcome: Objective cognitive performance (at the end of intervention).

Figure 5. Forest plot comparing cognitive interventions vs non-active controls and active controls in separate analyses for patients with subjective cognitive decline. Outcome: Objective cognitive performance (at the end of intervention).

Figure DS1. Forest plot comparing cognitive interventions vs control (all types). Outcome: Objective global cognitive performance (at the end of intervention).

For peer review only

For peer review only - http://bmjopen.bmj.com/site/about/guidelines.xhtml

REFERENCES

1. Jessen F, Amariglio RE, van BM, et al. A conceptual framework for research on subjective cognitive decline in preclinical Alzheimer's disease. *Alzheimers Dement* 2014;10(6):844-852.

2. Stone J, Pal S, Blackburn D, et al. Functional (Psychogenic) Cognitive Disorders: A Perspective from the Neurology Clinic. *J Alzheimers Dis* 2015;48 Suppl 1:S5-S17.

3. Schmidtke K, Metternich B. Validation of two inventories for the diagnosis and monitoring of functional memory disorder. J Psychosom Res 2009; 67(3):245-251.

4. Elfgren C, Gustafson L, Vestberg S, et al. Subjective memory complaints, neuropsychological performance and psychiatric variables in memory clinic attendees: a 3-year follow-up study. Arch Gerontol Geriatr 2010;51(3):e110-e114.

5. Alladi S, Arnold R, Mitchell J, et al. Mild cognitive impairment: applicability of research criteria in a memory clinic and characterization of cognitive profile. Psychol Med 2006;36(4):507-515.

6. Wahlund LO, Pihlstrand E, Jonhagen ME. Mild cognitive impairment: experience from a memory clinic. Acta Neurol Scand Suppl 2003;179:21-24.

7. Vraamark ET, Stokholm J, Hogh P, et al. Diagnostic profile of young and middle-aged memory clinic patients. Neurology 2002;59(8):1259-1262.

8. Jonker C, Geerlings MI, Schmand B. Are memory complaints predictive for dementia? A review of clinical and population-based studies. Int J Geriatr Psychiatry 2000;15(11):983-991.

9. Mitchell AJ, Beaumont H, Ferguson D, Yadegarfar M, Stubbs B. Risk of dementia and mild cognitive impairment in older people with subjective memory complaints: meta-analysis. Acta Psychiatr Scand 2014;130(6):439-451.

10. Amariglio RE, Becker JA, Carmasin J, et al. Subjective cognitive complaints and amyloid burden in cognitively normal older individuals. Neuropsychologia 2012;50(12):2880-2886.

11. Perrotin A, Mormino EC, Madison CM, et al. Subjective cognition and amyloid deposition imaging: a Pittsburgh Compound B positron emission tomography study in normal elderly individuals. Arch Neurol 2012;69(2):223-229.

12. Perrotin A, de Flores R, Lamberton F, et al. Hippocampal Subfield Volumetry and 3D Surface Mapping in Subjective Cognitive Decline. J Alzheimers Dis 2015;48 Suppl 1:S141-S150.

13. Peter J, Scheef L, Abdulkadir A, Boecker H, Heneka M, Wagner M et al. Gray matter atrophy pattern in elderly with subjective memory impairment. Alzheimers Dement 2014;10(1):99-108.

14. Hessen E, Eckerstrom M, Nordlund A, Selseth A, I, Stalhammar J, Bjerke M et al. Subjective Cognitive Impairment Is a Predominantly Benign Condition in Memory Clinic Patients Followed for 6 Years: The Gothenburg-Oslo MCI Study. Dement Geriatr Cogn Dis Extra 2017;7(1):1-14.

For peer review only - http://bmjopen.bmj.com/site/about/guidelines.xhtml

15. Mendonca MD, Alves L, Bugalho P. From Subjective Cognitive Complaints to Dementia: Who is at Risk?: A Systematic Review. Am J Alzheimers Dis Other Demen 2016;31(2):105-114.

16. Prince M, Comas-Herrera A, Knapp M et al. Improving healthcare for people living with dementia. Coverage, Quality and costs now and in the future. World Alzheimer Report, 2016. Available from www.alz.co.uk/research/WorldAlzheimerReport2016.pdf. Accessed on 20/12/17

17. Stern Y. Cognitive reserve in ageing and Alzheimer's disease. Lancet Neurol 2012;11(11):1006-1012.

18. Sohlberg MM, Mateer CA. Cognitive rehabilitation: An integrative neuropsychological approach. New York, Guilford 2001

19. Metternich B, Kosch D, Kriston L, et al. The effects of nonpharmacological interventions on subjective memory complaints: a systematic review and meta-analysis. Psychother Psychosom 2010;79(1):6-19.

20. Fleming SM, Dolan RJ. The neural basis of metacognitive ability. Philos Trans R Soc Lond B Biol Sci 2012;367(1594):1338-1349.

21. Metternich B, Schmidtke K, Hull M. How are memory complaints in functional memory disorder related to measures of affect, metamemory and cognition? J Psychosom Res 2009;66(5):435-444.

22. Canevelli M, Adali N, Tainturier C, et al. Cognitive interventions targeting subjective cognitive complaints. Am J Alzheimers Dis Other Demen 2013;28(6):560-567.

23. Smart CM, Karr JE, Areshenkoff CN, Rabin LA, Hudon C, Gates N et al. Non-Pharmacologic Interventions for Older Adults with Subjective Cognitive Decline: Systematic Review, Meta-Analysis, and Preliminary Recommendations. Neuropsychol Rev 2017;27(3):245-257.

24. Oh SJ, Seo S, Lee JH, et al. Effects of smartphone-based memory training for older adults with subjective memory complaints: a randomized controlled trial. Aging Ment Health 2017;1-9.

25. Pereira-Morales AJ, Cruz-Salinas AF, Aponte J, et al. Efficacy of a computer-based cognitive training program in older people with subjective memory complaints: a randomized study. Int J Neurosci 2018;128(1):1-9.

26. Huntley JD, Gould RL, Liu K, et al. Do cognitive interventions improve general cognition in dementia? A meta-analysis and meta-regression. BMJ Open 2015;5(4):e005247.

27. Cooper C, Li R, Lyketsos C, Livingston G. Treatment for mild cognitive impairment: systematic review. Br J Psychiatry 2013;203(3):255-264.

28. Rapaport P, Livingston G, Murray Jet al. Systematic review of the effective components of psychosocial interventions delivered by care home staff to people with dementia. BMJ Open 2017;7(2):e014177.

For peer review only - http://bmjopen.bmj.com/site/about/guidelines.xhtml

29. Borenstein M, Hedges LV, Higgins JPT, et al. Introduction to Meta-Analysis. Chichester, John Wiley & Sons, Ltd 2009.

30. Hoogenhout EM, de Groot RH, van der Elst W, et al. Effects of a comprehensive educational group intervention in older women with cognitive complaints: a randomized controlled trial. Aging Ment Health 2012;16(2):135-144.

31. Metternich B, Schmidtke K, Harter M, et al. [Development and evaluation of a group therapy for functional memory and attention disorder]. Psychother Psychosom Med Psychol 2010; 60(6):202-210.

32. van Hooren SA, Valentijn SA, Bosma H, et al. Effect of a structured course involving goal management training in older adults: A randomised controlled trial. Patient Educ Couns 2007;65(2):205-213.

33. Smart CM, Segalowitz SJ, Mulligan BP, et al. Mindfulness Training for Older Adults with Subjective Cognitive Decline: Results from a Pilot Randomized Controlled Trial. J Alzheimers Dis 2016;52(2):757-774.

34. Andrewes DG, Kinsella G, Murphy M. Using a memory handbook to improve everyday memory in community-dwelling older adults with memory complaints. Exp Aging Res 1996;22(3):305-322.

35. Barnes DE, Santos-Modesitt W, Poelke G, et al. The Mental Activity and eXercise (MAX) trial: a randomized controlled trial to enhance cognitive function in older adults. JAMA Intern Med 2013;173(9):797-804.

36. Cohen-Mansfield J, Cohen R, Buettner L, et al. Interventions for older persons reporting memory difficulties: a randomized controlled pilot study. Int J Geriatr Psychiatry 2015;30(5):478-486.

37. Kwok TC, Bai X, Li JC, et al. Effectiveness of cognitive training in Chinese older people with subjective cognitive complaints: a randomized placebo-controlled trial. Int J Geriatr Psychiatry 2013;28(2):208-215.

38. Pike KE, Ong B, Clare L, et al. Face-name memory training in subjective memory decline: how does office-based training translate to everyday situations? Neuropsychol Dev Cogn B Aging Neuropsychol Cogn 2017;1-29.

39. Scogin F, Storandt M, Lott L. Memory-skills training, memory complaints, and depression in older adults. J Gerontol 1985;40(5):562-568.

40. Tsai AY, Yang MJ, Lan CF, et al. Evaluation of effect of cognitive intervention programs for the community-dwelling elderly with subjective memory complaints. Int J Geriatr Psychiatry 2008;23(11):1172-1174.

41. Valentijn SA, van Hooren SA, Bosma H, et al. The effect of two types of memory training on subjective and objective memory performance in healthy individuals aged 55 years and older: a randomized controlled trial. Patient Educ Couns 2005;57(1):106-114.

For peer review only - http://bmjopen.bmj.com/site/about/guidelines.xhtml

42. Youn JH, Lee JY, Kim S, et al. Multistrategic memory training with the metamemory concept in healthy older adults. Psychiatry Investig 2011;8(4):354-361.

43. Small GW, Silverman DH, Siddarth P, et al. Effects of a 14-day healthy longevity lifestyle program on cognition and brain function. Am J Geriatr Psychiatry 2006;14(6):538-545.

44. Beck SM, Ruge H, Schindler C, et al. Effects of Ginkgo biloba extract EGb 761(R) on cognitive control functions, mental activity of the prefrontal cortex and stress reactivity in elderly adults with subjective memory impairment - a randomized double-blind placebo-controlled trial. Hum Psychopharmacol 2016;31(3):227-242.

45. Boespflug EL, McNamara RK, Eliassen JC, et al. Fish Oil Supplementation Increases Event-Related Posterior Cingulate Activation in Older Adults with Subjective Memory Impairment. J Nutr Health Aging 2016;20(2):161-169.

46. Brautigam MR, Blommaert FA, Verleye G, et al. Treatment of age-related memory complaints with Ginkgo biloba extract: a randomized double blind placebo-controlled study. Phytomedicine 1998;5(6):425-434.

47. Zhu J, Shi R, Chen S, et al. The Relieving Effects of BrainPower Advanced, a Dietary Supplement, in Older Adults with Subjective Memory Complaints: A Randomized, Double-Blind, Placebo-Controlled Trial. Evid Based Complement Alternat Med 2016:7898093.

48. Sullivan GM, Feinn R. Using Effect Size—or Why the P Value Is Not Enough. J Grad Med Educ 2012;4(3):279-282.

49. Bures V, Cech P, Mikulecka J, et al. The effect of cognitive training on the subjective perception of well-being in older adults. PeerJ 2016;4:e2785.

50. Woods B, Thorgrimsen L, Spector A, et al. Improved quality of life and cognitive stimulation therapy in dementia. Aging Ment Health 2006;10(3):219-226.

51. Morris S. Estimating Effect Sizes From Pretest-Posttest-Control Group Designs. Organ. Res. Meth 2008;11(2):364-386.

52. Hedges LV, Olkin I. Statistical methods for meta-analysis. New York, Academic Press 1985.

53. DerSimonian R, Laird N. Meta-analysis in clinical trials. Control Clin Trials 1986; 7(3):177-188.

For peer review only

For peer review only - http://bmjopen.bmj.com/site/about/guidelines.xhtml

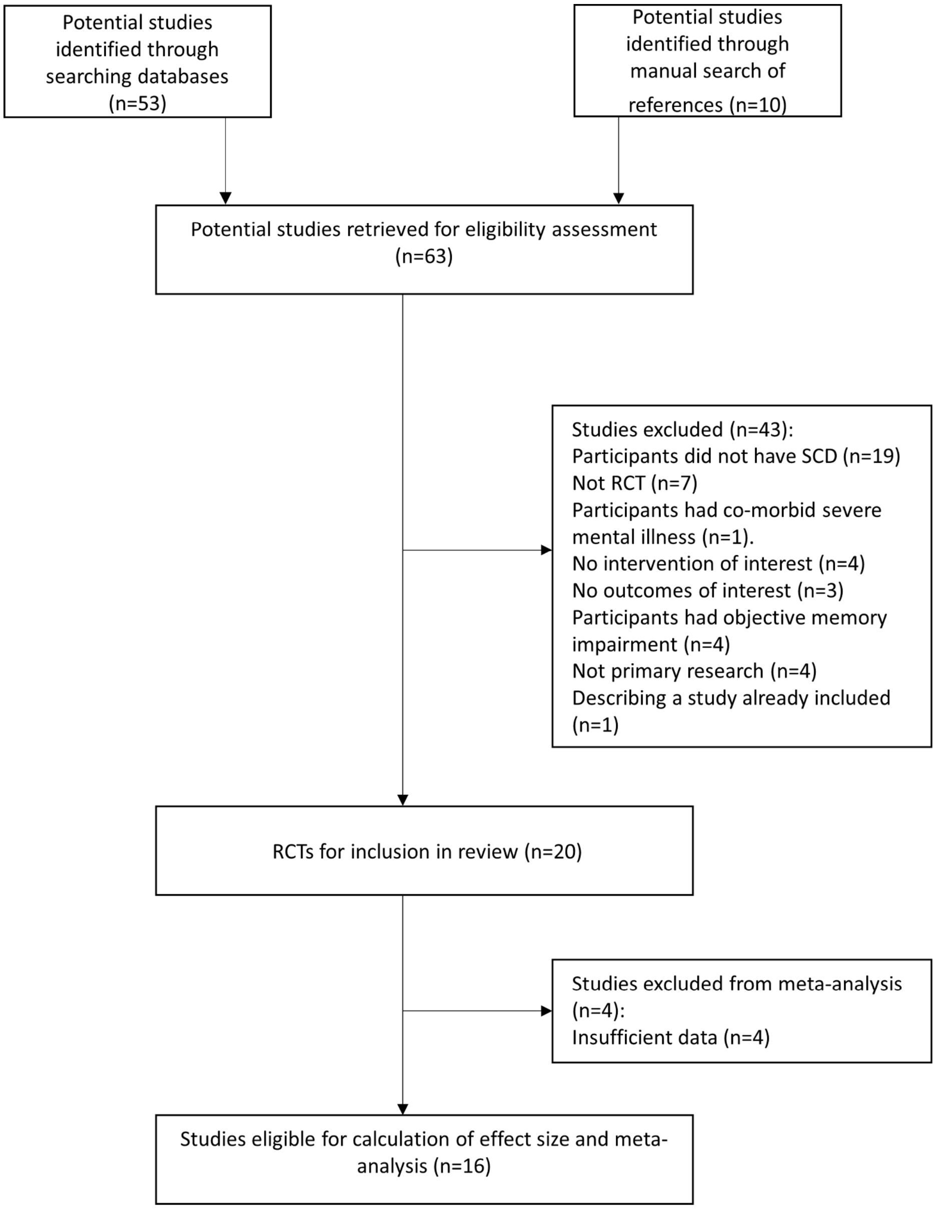

Figure 1. Details of search strategy. RCT, Randomised control trial.

175x227mm (300 x 300 DPI)

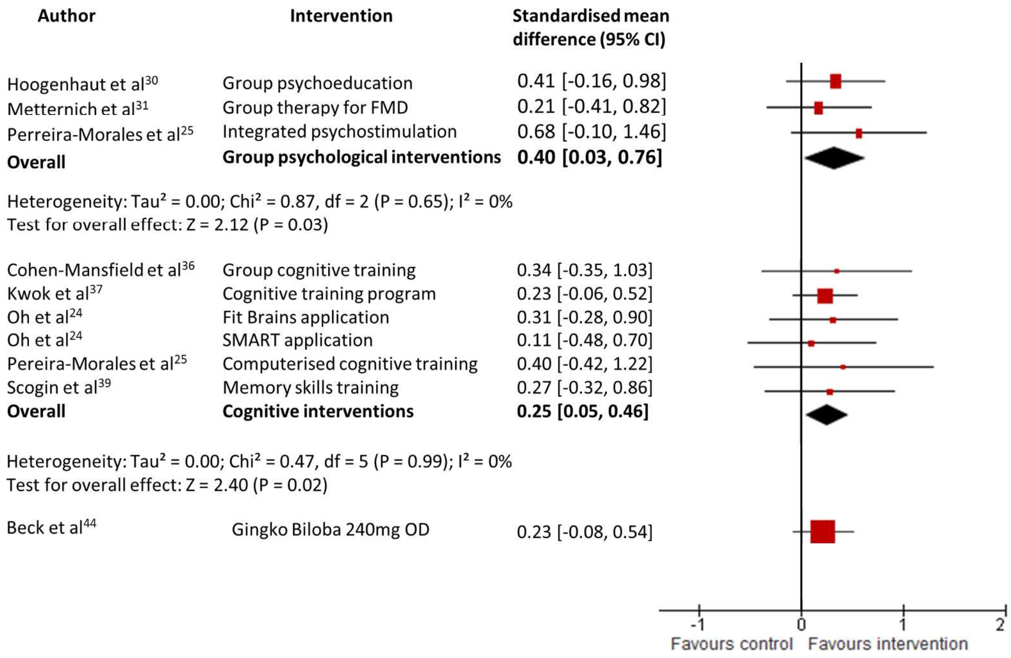

Figure 2. Forest plot comparing interventions (all types) vs control (all types) for patients with subjective cognitive decline. Outcome: Psychological well-being (at the end of intervention).

180x115mm (300 x 300 DPI)

For peer review only

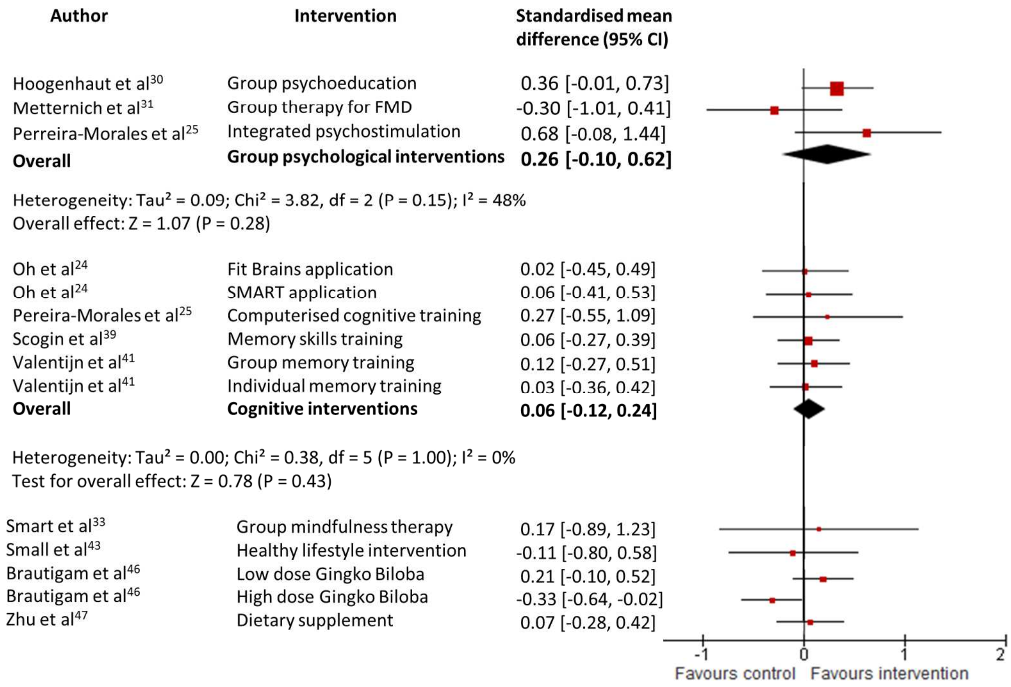

Figure 3. Forest plot comparing interventions (all types) vs control (all types) for patients with subjective cognitive decline. Outcome: Metacognitive ability (at the end of intervention).

181x121mm (300 x 300 DPI)

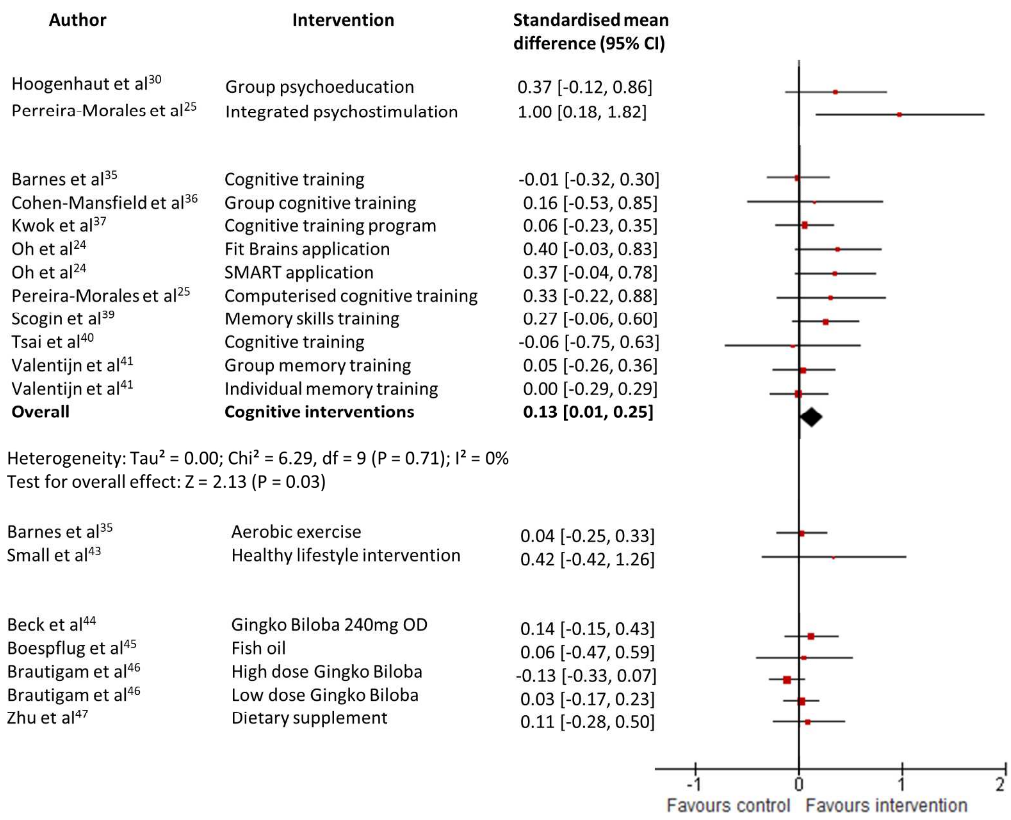

Figure 4. Forest plot comparing interventions (all types) vs control (all types) for patients with subjective cognitive decline. Outcome: Objective cognitive performance (at the end of intervention).

181x145mm (300 x 300 DPI)

For peer review only - http://bmjopen.bmj.com/site/about/guidelines.xhtml

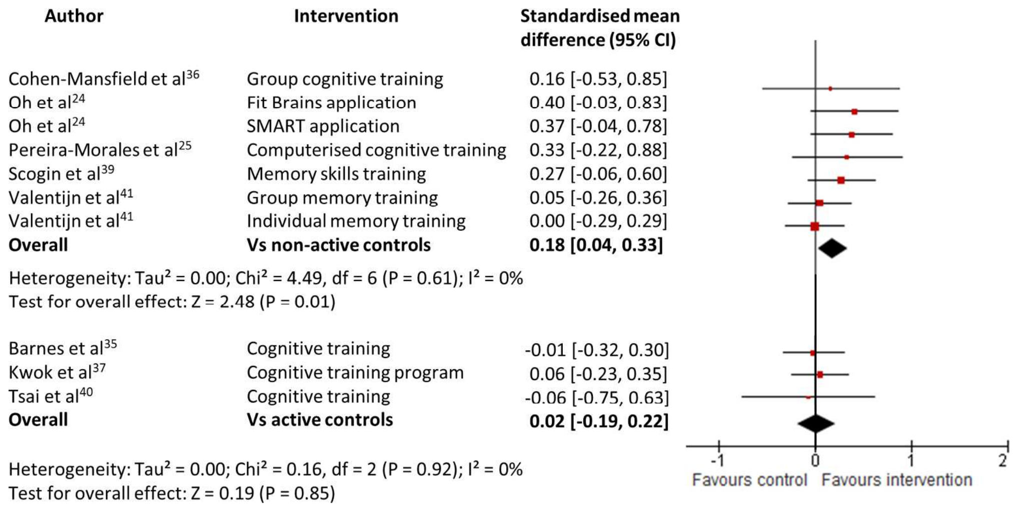

Figure 5. Forest plot comparing cognitive interventions vs non-active controls and active controls in separate analyses for patients with subjective cognitive decline. Outcome: Objective cognitive performance (at the end of intervention).

177x87mm (300 x 300 DPI)

review only

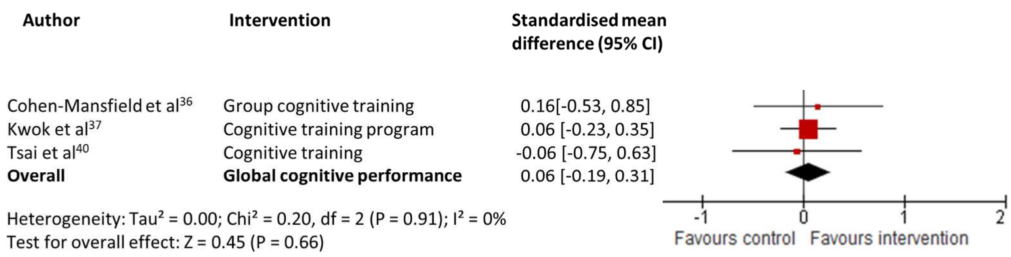

Figure DS1. Forest plot comparing cognitive interventions vs control (all types). Outcome: Objective global cognitive performance (at the end of intervention).

182x43mm (300 x 300 DPI)

For peer review only

Supplementary Appendix 1

Assessment of Trial Quality

The following questions were considered for each included study:

1. Were participants randomised independently to treatment and control groups?
2. Were participants and clinicians 'blinded', where possible, to treatment allocation?
3. Was incomplete outcome data dealt with using 'intention to treat' analysis?
4. Were all participants followed up and data collected in the same way?
5. Was a power calculation carried out, a priori, based on at least one of our outcomes of interest?

Any disagreements between raters (RB, AB and JH) were resolved through discussion with a fourth author (RH). Each checklist item above scored 1 point, therefore each study could score between 0 and 5 for validity. As with previous work[28] studies which independently randomised participants to treatment and control groups, used intention to treat analysis to manage incomplete outcome data and followed up patients in the same way (points 1, 3 and 4 above) were deemed to be high quality.

Statistical methods

Effect size calculation

Effect sizes were calculated using RevMan software. Standardised mean differences were calculated using Hedges' adjusted g.

Pre-intervention standard deviations were used as these are most likely to be comparable across studies and therefore provide the most accurate estimate of effect size.[51]

The Hedges' adjusted g formula[52] used in RevMan is as follows:

$$g= [M_{post\ intervention} – M_{post\ control}/SD_{pre\text{-}pooled}]*[1- 3/(4N\text{-}9)]$$

Where $N= n_{intervention\ group} + n_{control\ group}$

and

$$SD_{pre\text{-}pooled} = \sqrt{} [((n_{intervention\text{-}1})\ SD_{pre\ intervention}^2 + (n_{control\text{-}1})\ SD_{pre\ control}^2)/ N\text{-}2]$$

Meta-analyses

Meta-analyses were performed using RevMan software. A random effects method as described by DeSirmonian and laird[53] was used. This adjusts standard errors of the effect sizes in each study to account for the heterogeneity for intervention effects observed between different studies.

The pooled effect size of each meta-analysis was calculated by attributing a weight to the average effect size in each study according to sample size. The z statistic was used to evaluate whether the pooled effect size was significantly different to no effect.

Heterogeneity was quantified using the I^s statistic.

Composite measure calculation

For peer review only

Composite scores were calculated where a study reported multiple outcomes falling within a particular outcome domain (e.g. objective cognitive performance). This approach was pragmatic in allowing one score to represent each intervention in the meta-analysis regardless of the number of outcomes reported. In turn this prevents more weight being given to studies with multiple outcomes.[54] The variance of the sum of variables was calculated as described below.

Using the example of a study with two relevant outcomes, there will be two effect sizes, namely y_1 and y_2. The overall mean effect size for the composite measure will be:

$\bar{y}= ½ (y_1 + y_2)$

The variance of this mean is calculated as follows:

$V_{\bar{y}}= ¼ (V_{Y1} + V_{y2} + 2r*\sqrt{V_{Y1}}*\sqrt{V_{y2}})$,

where r is the correlation coefficient describing to what extent y_1 and y_2 co-vary.

If the correlation is set at 0, the outcomes are essentially treated as independent of each other and if the correlation is set at 1, the variance is an average of each outcome's variance. The former will lead to an underestimation of the variance and overestimation of precision while the latter will have the opposite effect. Consequently, in the absence of existing literature to identify a suitable correlation, we reported composite effect sizes calculated using a correlation of 0.5.

Supplementary appendix 2

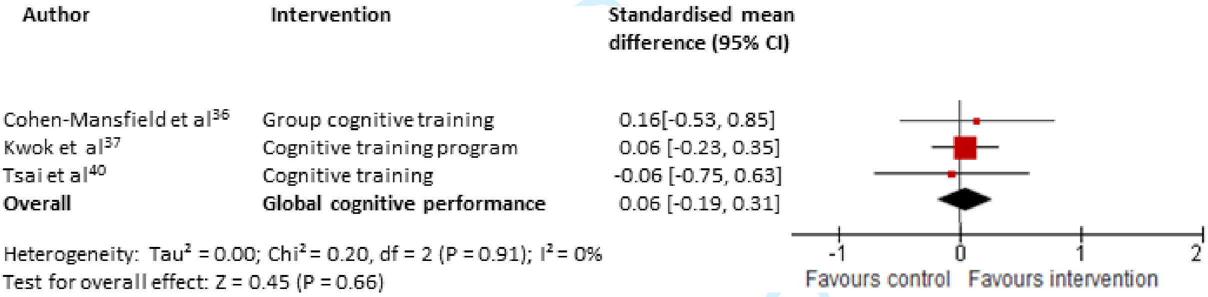

Fig. DS1 Forest plot comparing cognitive interventions vs control (all types). Outcome: Objective global cognitive performance (at the end of intervention).

For peer review only

For peer review only

Table DS1. Characteristics and validity of included studies

Study	Recruitment Setting	Inclusion criteria	Treatment group		Control group		Outcomes	Key findings: treatment vs control group, measured at end of intervention unless stated	Validity (answer to questions 1-5 in methods)				
			Treatment	n	Treatment	n			1	2	3	4	5
*Andrewes et al	Volunteers in the community	SMC; MDRS score > 123; Age 60-70 years	Memory training- participants provided with a handbook containing information and practice exercises to aid memory.<br><br>4 weeks	20	Non-active control- pamphlets providing information about three mnemonics.<br><br>4 weeks	20	Memory strategy knowledge questionnaire, Memory Strategy Use Questionnaire, memory diary.<br><br>Face-Name test, Prospective memory,	No differences in prospective memory tasks.<br><br>Significant improvement in face-name recall in memory training group.<br><br>No difference in cognitive measures at 4-months follow up.	y	y	y	y	n
Barnes et al	Volunteers in the community	SMC; Age >65 years	Mental activity performed on computers to enhance auditory and visual processing.<br><br>60 mins, 3 days a week for 12 weeks.	32	Active control- watched DVDs of educational lectures, on art, history and science.<br><br>60 mins, 3 days a week for 12 weeks	31	RAVLT, EFT, DSST, TMT-A, TMT-B, UFOV, Verbal fluency	Visuospatial function improved significantly for treatment group; improved global cognitive functioning in both groups but no significant inter-group differences.	y	y	y	y	y

For peer review only

Barnes et al	Volunteers in the community	SMC; Age>65 years	Aerobic exercise aiming to increase heart rates to 60-75% of participants' maximum.<br><br>60 mins, 3 days a week for 12 weeks	32	Active control-without aerobic exercise portion. Aim was not to increase resting heart rate-<br><br>60 mins, 3 days a week for 12 weeks	31	RAVLT, EFT, DSST, TMT-A, TMT-B, UFOV, Verbal fluency	Improved global cognitive functioning in both groups but no significant inter-group differences.	y	y	y	y	y
Beck et al	Volunteers in the community	SMI as diagnosed by the PRMQ.	Gingko Biloba 240mg OD<br><br>8 weeks	43	Placebo<br><br>8 weeks	32	Task set shifting, delayed response task, prospective memory task, Go-NoGo task,<br><br>MDBF subscales for good/bad mood; alertness/fatigue; calmness/restlessness pre and post TSST	Significant improvement in task-set shifting (executive function) in treatment group.	y	y	y	y	n
Bosepflug et al	Volunteers in the community	SMC	Fish oil (1.6g eicosapentaenoic acid (EPA) and 0.8g	11	Placebo- corn oil<br><br>24 weeks	10	Working memory task	Significant improvement of working memory in the 2-back	y	y	_	y	n

			docosahexaenoic acid (DHA)) daily.<br><br>24 weeks.					condition. In the treatment group					
Brautigham et al	Volunteers in the community	SCD; non-institutionalised; Age 55-86 years	Gingko 40 drops (1.9mls) undiluted daily<br><br>24 weeks	72	Placebo<br><br>24 weeks	82	15 Words of Rey (part 1 and 2); Benton test of visual retention-revised; EMCT	Statistically significant improvement in visual memory that was dose-dependent	y	y	n	y	n
Brautigham et al	Volunteers in the community	SCD; non-institutionalised; Age 55-86 years	Gingko 40 drops (1.9mls) 1:1 dilution<br><br>24 weeks	72	Placebo<br><br>24 weeks	82	15 Words of Rey (part 1 and 2); Benton test of visual retention-revised; EMCT	Statistically significant improvement in visual memory that was dose-dependent	y	y	n	y	n
Cohen-Mansfield et al	Volunteers in the community	SMC MMSE >24; Hebrew language; Age>65 years	Cogntive training course-participant centred, delivered in a book club format with memory, reasoning and speed of processing being the main foci.	15	Waiting list control	28	UCLA loneliness scale<br><br>Global cognitive score;	Significantly improved global cognitive score and significant reduction in loneliness and self-reported memory difficulties.	y	y	n	y	n

For peer review only

			10 weeks										
Cohen-Mansfield et al	Volunteers in the community	SMC MMSE >24; Hebrew language; Age>65 years	Participant-centered course-delivery of memory, cognitive and organisational strategies. Cognitive behavioural principles used whereby individuals had mistaken conceptions of memory problems and self-worth challenged.<br><br>10 weeks	14	Non-active control	12	UCLA loneliness scale<br><br>Global cognitive score;	Improvement in cognitive scores from pre to post intervention. Trend towards reduction in loneliness. No significant difference compared to control.	y	y	n	y	n
Cohen-Mansfield et al	Volunteers in the community	SMC MMSE >24; Hebrew language; Age>65 years	Health promotion- topics included health behaviours, dementia, cognitive activities, relationships, depression and coping, home and travel safety,	15	Non-active control	12	UCLA loneliness scale<br><br>Global cognitive score;	Improvement in cognitive scores from pre to post intervention. Trend towards reduction in loneliness. No significant difference compared to control.	y	y	n	y	n

For peer review only

			recreation and leisure, medications and health care providers, physical activity and lifelong learning.<br><br>10 weeks										
Hoogenhaut et al	Volunteers in the community	SCD; women; Age 60-75 years	Psychoeducation-information delivered on subjective cognitive difficulties, ageing and internal/external strategies.<br><br>Eight 1.5 hour sessions delivered over 4 weeks.	30	Waiting list control	30	MMI<br><br>Psychological well-being quotient (CES-D, NCQ, SF-36);<br><br>Memory quotient based on VVLT; Executive Functioning and Speed Quotient- (CST, SCWT, LDST)	Participants in the experimental group reported fewer emotional reactions towards cognitive functioning post-intervention.<br><br>No significant differences between groups for psychological well-being, memory or executive function.	y	y	y	y	N

For peer review only

Kwok et al	Volunteers from community centres	SMC; Age >60 years; no physical or psychiatric co-morbidity associated with cognitive impairment; >22 on MMSE	Active mind training programme- education on memory decline and dementia together with cognitive training.<br><br>1 hour, once a week for 8 weeks	86	Active control- usual group activities provided by community centre such as daily discussion about the news, outings and monthly gatherings.	90	Total adjusted CDRS; CDRS scores for attention, Initiation, construction Conceptualisation, SF12-Mental	Significant improvement in mental health subscore on SF 12 questionnaire.<br><br>Significant improvement in overall global cognition with conceptualisation being most marked.	y	y	n	y	Y
Metternich et al	Memory Clinic	Meet criteria for diagnosis of FMD; Age <69; IQ>80;	Psychoeducation, cognitive restructuring, stress management, relaxation and mindfulness techniques.	18	Waiting list control	18	SCL-90 R GSI; PSQ<br><br>MIA	Significant improvement in MSE at 3 month follow up post intervention. No significant difference immediately after the intervention.	y	y	y	y	y
Oh et al	Volunteers in the community	SMC; Smartphone owner; K-MMSE >23	SMART – a smartphone application developed to improve the user's attention and working memory. Tasks have three difficulty levels.	18	Waiting list control	16	MMQ<br><br>CES-D; STAI-S<br><br>MDS	Significant improvement in working memory quotient and auditory-working memory.	y	y	n	y	n

For peer review only

			15-20 minutes daily for 5 days a week for 8 weeks										
Oh et al	Volunteers in the community	SMC; Smartphone owner; K-MMSE >23	Fitbrains-smartphone application providing cognitive training. Three tasks within each session with thirty sessions in total. Recommendation to complete one session daily. Each task was completed twice to increase training time.<br><br>Unclear over what duration training was undertaken but at least 30 days over an 8 week period.	19	Waiting list control	16	MMQ<br><br>CES-D; STAI-S<br><br>MDS	Significant improvement in contentment on MMQ	y	y	n	y	n
Pereira-Morales et al	Volunteers from community centres	MMSE>21; no diagnosis of MCI/ dementia;	Integrated psycho-stimulation programme (IPP):	17	Non-active control-Information brochure on how to reduce risks of	11	SMCQ<br><br>STAI	Significant improvement in metamemory and anxiety in IPP	-	n	-	y	n

For peer review only

		intact ADLs; no significant co-morbid psychiatric or neurological condition.	Cognitive training, progressive relaxation exercises, meta-memory activities and group discussions to engender socialisation..<br><br>90 mins, 4 times a week for 8 weeks		developing MCI/ dementia		Grober and Buschke short/long term memory; Stroop; WAIS	group compared to baseline.					
Pereira-Morales et al	Volunteers from community centres	MMSE>21; no diagnosis of MCI/ dementia; intact ADLs; no significant co-morbid psychiatric or neurological condition.	Computerised cognitive training (CCT).<br>60 mins, 4 times a week for 8 weeks.	12	Non-active control- Information brochure on how to reduce risks of developing MCI/ dementia	11	SMCQ<br><br>STAI<br><br>Grober and Buschke short/long term memory; Stroop; WAIS	Significant improvement in cognition as compared to baseline.	-	n	-	y	n
*Pike et al	Volunteers from community centres	SMC; Age>60; community dwelling; independent ADLs,; English language	Face-name memory training in an office based setting. Either allocated to receive training based on semantic association (SA)	22 (SA)<br><br>25 (SR)	No training	21<br><br>21	Cued/uncued recall.<br><br>MMQ-contentment MCI-memory control beliefs	Spaced retrieval improved recall for cued and uncued names, whilst semantic association improved only recall for cued names. Neither	Y	n	n	n	n

For peer review only

			or spaced retrieval (SR).<br><br>One off 1.5 hour session followed by 2 booster phone calls.					were statistically significant.<br><br>No improvement with either intervention in naturalistic setting when assessed two months later.					
Scogin et al	Volunteers in the community.	SMD; Age > 60;	Self-administered cognitive training from a manual.<br><br>Sixteen one hour study sessions.<br><br>Once weekly phone calls from first author to ensure compliance	20	Waiting list control	27	Memory recall tasks; Benton Visual Retention Test.<br><br>Metamemory Questionnaire.<br><br>Zhung depression scale.	No significant difference in composite measure of metacognition or objective cognitive performance or in depression scores<br><br>No differences in memory complaint scores or depression scores.	n	n	n	y	n
Small et al	Volunteers in the community.	SMI; Age 35-69; absence of severe psychiatric or neurological illness; absence of MCI; not	14-day Healthy lifestyle programme based on mental and physical exercises, healthy diet, and stress reduction techniques.	8	Non-active control.<br><br>Continued lifestyle as usual.<br><br>2 weeks	9	Buschke Fuld Selective Reminding Test; COWAT<br><br>MFQ	Verbal fluency improved significantly in intervention group but no significant difference between groups.	n	n	n	y	n

For peer review only

		taking psychotropic medication	2 weeks					No statistically significant differences between groups.					
Smart et al	Volunteers in the community	SCC; Age 65-85; English language; no significant neurological disease; MMSE ≥25;tolerate MRI;	Mindfulness based therapy<br><br>Eight 2 hour long, once weekly group sessions	8	Active control-psychoeducation<br><br>Five 2 hour long, once weekly group sessions	6	Cognitive complaints index – composite score made from cognitive subscales of GDS, MCQ and MIA<br><br>FFMQ-A	P3 ERP amplitude significantly increased.<br><br>No significant Improvements in self-reported subjective memory complaints, mindful attention measures.	-	y	-	y	n
Tsai et al	Volunteers from community setting.	SMC; Age >55; CDR score 0; Independent ADLs; Chinese language	Cognitive training which included strategies and skills to improve cognitive performance. Also taught meditation and some psychoeducation.<br><br>2 hours, twice weekly for 5 weeks.	14	Active control-cognitive stimulation Which included problem solving exercises and quizzes.<br><br>1.5 hours, once weekly for 8 weeks.	11	ADAS-Cog; MMSE; SRT Clock Drawing Test	Statistically significant improvements in MMSE and ADAS-Cog in both groups at the end of the intervention and 6 months follow up but no significant difference between groups.	n	n	n	y	n

Valentijn et al	Volunteers from community setting.	SMC; Age>55; Dutch language; >24 on MMSE; mobile,	Collective memory training including internal and external memory strategies as well as psychoeducation.<br><br>2 hour weekly session for 8 weeks	53	Waiting list control	43	VVLT; Short story test; CFQ<br><br>MIA	Significant improvement in recall.<br><br>Significant improvement on MIA change and anxiety subscale score.	y	n	y	n	n
Valentijn et al	Volunteers in the community	SMC; Age >55; Dutch language; MMSE >24; mobile	Individual training – subjects given "Memory support" book with the content described in the same order as above. 8-week time period (called 4 weeks through to ensure compliance with the training)	43	Waiting list control	43	VVLT; Short story test; CFQ<br><br>MIA	Significant improvement in recall.<br><br>Significant improvement in MIA anxiety scores.	y	n	y	n	n
*Van Hooren et al	Volunteers in the community.	SCC; Age>55; MMSE≥24; Dutch language; travel independently; absence of	Goal management training- structured and interactive intervention to teach individuals	38	Waiting list control	31	SCWT<br><br>CFQ<br><br>SCL-90	Significant reduction in anxiety scores.<br><br>Significant reduction in annoyance towards cognitive failures.	y	n	y	n	n

For peer review only

		psychiatric or neurological co-morbidity.	a strategy to improve planning activities and to structure intentions. Additionally, there was a psychoeducation component about cognitive ageing.<br><br>12 sessions each 1.5 hours long delivered over 6 weeks (1 individual and 11 group sessions)					Significant reduction in number of executive failures reported.					
*Youn et al	Memory clinic	SMC; Age > 55; excluded if serious psychiatric or medical co-morbidity	Cognitive training (focus on metamemory)<br><br>10 week programme, of 90 min 1x weekly sessions	20	Waiting list control	20	SRFT; Elderly verbal learning test; DST; VST; Phonemic fluency test; Categorical fluency test<br><br>SMCQ<br><br>GDS	Significant improvements on short and long term recall, SRFT recognition, VST (forward), categorical fluency.	y	Y	n	y	n

For peer review only

Zhu et al	Volunteers in the community	SCC; Age ≥50 years, not on psychotropic medication, , no severe psychiatric or medical conditions	Brain Power Advanced supplement.<br><br>2 capsules with meals for 12 weeks.	47	Non-active control- placebo tablets.<br><br>2 capsules with meals for 12 weeks.	51	Visual, auditory and short-term memory<br><br>Subjective memory loss and subjective attentional deficits.	No significant difference between in subjective memory loss or subjective attentional deficit scores.	Y	y	n	y	n

SMC, Subjective memory complaints; MDRS, Mattis Dementia Rating Scale; RAVLT, Rey Auditory Verbal Learning Test; EFT, Eriksen Flanker Test; DSST, Digit Symbol Substitution Test; TMT-A, Trail Making Test A; TMT-B, Trail Making Test B; UFOV, Useful field of view; TSST, Trier Social Stress Test; SMI, subjective memory impairment; PRMQ, Prospective and Retrospective Memory Questionnaire; EMCT, Expanded Mental Control Test; SCD, Subjective Cognitive Decline; MMI, Maastrict Metacognition Inventory; CES-D, Centre of Epidemiologic Studies Depression scale; NCQ, Neurovegetative Complaints Questionnaire; SF-36, Short-Form Health Survey; CST, Concept Shifting Test; SCWT, Stroop Color-Word Test; LDST, Letter Digit Substitution; VVLT, Visual Verbal Learning Test; SF12, 12-Item Short Form Health Survey; CDRS, Chinese version of Mattis Dementia Rating Scale; SCL-90 R GSI, Symptom Checklist 90-R; MIA, Metamemory in Adulthood Questionnaire; PSQ, Perceived Stress Questionnaire; FMD, Functional Memory Disorder; WAIS, Wechsler Adult Intelligence Scale; STAI, State-Trait Anxiety Inventory; SMCQ, Subjective Memory Complaints Questionnaire; MMQ, Multifactorial Memory Questionnaire; MDS, Memory Diagnostic System; MCI, Mild Cognitive Impairment; SRT, Selective Reminding Test; MMSE, Mini-Mental State Examination; CDR, Clinical Dementia Rating; ADAS-cog, Alzheimer's disease Assessment Scale-Cognition; CFQ, Cognitive Failure Questionnaire; SRFT, Simple-Rey figure test; DST, Digit Span Test; VST, Visual Span Test; MCQ, Memory Complaints Questionnaire; FFMQ-A, five facet mindfulness questionnaire- "mindful attention" subscales; COWAT, Controlled oral word association test; MFQ, Memory Function Questionnaire; SMI, Subjective Memory impairment.
***Not included in meta-analysis**

For peer review only

PRISMA 2009 Checklist

Section/topic	#	Checklist item	Reported on page #
**TITLE**			
Title	1	Identify the report as a systematic review, meta-analysis, or both.	1
**ABSTRACT**			
Structured summary	2	Provide a structured summary including, as applicable: background; objectives; data sources; study eligibility criteria, participants, and interventions; study appraisal and synthesis methods; results; limitations; conclusions and implications of key findings; systematic review registration number.	2
**INTRODUCTION**			
Rationale	3	Describe the rationale for the review in the context of what is already known.	4
Objectives	4	Provide an explicit statement of questions being addressed with reference to participants, interventions, comparisons, outcomes, and study design (PICOS).	4
**METHODS**			
Protocol and registration	5	Indicate if a review protocol exists, if and where it can be accessed (e.g., Web address), and, if available, provide registration information including registration number.	1
Eligibility criteria	6	Specify study characteristics (e.g., PICOS, length of follow-up) and report characteristics (e.g., years considered, language, publication status) used as criteria for eligibility, giving rationale.	5
Information sources	7	Describe all information sources (e.g., databases with dates of coverage, contact with study authors to identify additional studies) in the search and date last searched.	5
Search	8	Present full electronic search strategy for at least one database, including any limits used, such that it could be repeated.	5
Study selection	9	State the process for selecting studies (i.e., screening, eligibility, included in systematic review, and, if applicable, included in the meta-analysis).	5
Data collection process	10	Describe method of data extraction from reports (e.g., piloted forms, independently, in duplicate) and any processes for obtaining and confirming data from investigators.	5
Data items	11	List and define all variables for which data were sought (e.g., PICOS, funding sources) and any assumptions and simplifications made.	5
Risk of bias in individual studies	12	Describe methods used for assessing risk of bias of individual studies (including specification of whether this was done at the study or outcome level), and how this information is to be used in any data synthesis.	5, supplementary data files 1 and 2
Summary measures	13	State the principal summary measures (e.g., risk ratio, difference in means).	5, supplementary

For peer review only - http://bmjopen.bmj.com/site/about/guidelines.xhtml

PRISMA 2009 Checklist

			data file 1
Synthesis of results	14	Describe the methods of handling data and combining results of studies, if done, including measures of consistency (e.g., I^2) for each meta-analysis.	5, figures 2-5 supplementary data file 1

Section/topic	#	Checklist item	Reported on page #
Risk of bias across studies	15	Specify any assessment of risk of bias that may affect the cumulative evidence (e.g., publication bias, selective reporting within studies).	5, supplementary files 1 and 2
Additional analyses	16	Describe methods of additional analyses (e.g., sensitivity or subgroup analyses, meta-regression), if done, indicating which were pre-specified.	Not done
**RESULTS**			
Study selection	17	Give numbers of studies screened, assessed for eligibility, and included in the review, with reasons for exclusions at each stage, ideally with a flow diagram.	6, Figure 1
Study characteristics	18	For each study, present characteristics for which data were extracted (e.g., study size, PICOS, follow-up period) and provide the citations.	Supplementary data file 2
Risk of bias within studies	19	Present data on risk of bias of each study and, if available, any outcome level assessment (see item 12).	5, supplementary data files 1 and 2
Results of individual studies	20	For all outcomes considered (benefits or harms), present, for each study: (a) simple summary data for each intervention group (b) effect estimates and confidence intervals, ideally with a forest plot.	6-9, Figures 2-5, supplementary data file 2
Synthesis of results	21	Present results of each meta-analysis done, including confidence intervals and measures of consistency.	Figures 2-5
Risk of bias across studies	22	Present results of any assessment of risk of bias across studies (see Item 15).	Not done
Additional analysis	23	Give results of additional analyses, if done (e.g., sensitivity or subgroup analyses, meta-regression [see Item 16]).	Not done
**DISCUSSION**			
Summary of evidence	24	Summarize the main findings including the strength of evidence for each main outcome; consider their relevance to key groups (e.g., healthcare providers, users, and policy makers).	9-11

For peer review only - http://bmjopen.bmj.com/site/about/guidelines.xhtml

PRISMA 2009 Checklist

Limitations	25	Discuss limitations at study and outcome level (e.g., risk of bias), and at review-level (e.g., incomplete retrieval of identified research, reporting bias).	10-11
Conclusions	26	Provide a general interpretation of the results in the context of other evidence, and implications for future research.	11
**FUNDING**			
Funding	27	Describe sources of funding for the systematic review and other support (e.g., supply of data); role of funders for the systematic review.	12

From: Moher D, Liberati A, Tetzlaff J, Altman DG, The PRISMA Group (2009). Preferred Reporting Items for Systematic Reviews and Meta-Analyses: The PRISMA Statement. PLoS Med 6(7): e1000097. doi:10.1371/journal.pmed1000097

For more information, visit: **www.prisma-statement.org**.

For peer review only

For peer review only - http://bmjopen.bmj.com/site/about/guidelines.xhtml

BMJ Open

Interventions for Subjective Cognitive Decline: Systematic Review and Meta-analysis

Journal:	*BMJ Open*
Manuscript ID	bmjopen-2018-021610.R1
Article Type:	Research
Date Submitted by the Author:	03-May-2018
Complete List of Authors:	Bhome, Rohan; University College London, Division of Psychiatry<br>Berry, Alex; Camden and Islington NHS Foundation Trust<br>Huntley, Jonathan; University College London, Division of Psychiatry<br>Howard, R; University College London Division of Psychiatry,
**Primary Subject Heading**:	Mental health
Secondary Subject Heading:	Patient-centred medicine, Geriatric medicine, General practice / Family practice
Keywords:	Subjective cognitive decline, Metacognition, Group interventions, Psychologcial well-being, Systematic review

SCHOLARONE™
Manuscripts

Interventions for Subjective Cognitive Decline: Systematic Review and Meta-analysis

Title Page

Title: Interventions for Subjective Cognitive Decline: Systematic Review and Meta-analysis

First and corresponding author: Rohan Bhome, Division of Psychiatry, University College London, London, UK. rohan.bhome@ucl.ac.uk

2nd author: Alex J Berry, Camden and Islington Foundation Trust, London, UK

3rd author: Jonathan D Huntley, Division of Psychiatry, University College London, London, UK

Last author: Robert J Howard, Division of Psychiatry, University College London, London, UK

Word count: 4072 words

PROSPERO registration number: CRD42017079391

For peer review only

ABSTRACT

Objectives: This review provides a broad overview of the effectiveness of interventions for Subjective Cognitive Decline (SCD) in improving psychological well-being, metacognition and objective cognitive performance.

Methods: Databases including PubMed, Web of Science and Cochrane Systematic Reviews were searched up to August 2017 to identify randomised controlled trials (RCTs) evaluating interventions for SCD. Interventions were categorised as psychological, cognitive, lifestyle or pharmacological. Outcomes of interest included psychological well-being, metacognitive ability and objective cognitive performance. To assess the risk of bias, three authors independently rated study validity using criteria based on the Critical Appraisal Skills Programme. Random effects meta-analyses were undertaken where three or more studies investigated similar interventions and reported comparable outcomes.

Results: Twenty studies met inclusion criteria and 16 had sufficient data for inclusion in the meta-analyses. Of these, only seven were rated as being high quality. Group psychological interventions significantly improved psychological well-being (g=0.40, 95% CI 0.03-0.76; P=0.03) but the improvement they conferred on metacognitive ability was not statistically significant (g=0.26, 95% CI -0.22-0.73; P=0.28). Overall, cognitive training interventions led to a small, statistically significant improvement in objective cognitive performance (g=0.13, CI 0.01-0.25; P=0.03). However, the pooled effect sizes of studies using active control groups (g=0.02 CI -0.19-0.22; P=0.85) or reporting global cognitive measures (g=0.06, CI=-0.19-0.31; P=0.66) were non-significant.

Conclusions: There is a lack of high quality research in this field. Group psychological interventions improve psychological well-being and may also improve metacognition. A large, high quality study is indicated to investigate this further. There is no evidence to suggest that cognitive interventions improve global cognitive performance and the clinical utility of small improvements in specific cognitive domains is questionable. There is a lack of research considering lifestyle interventions and poor quality evidence for pharmacological interventions.

Strengths and Limitations of this study

This is a comprehensive systematic review, including meta-analysis, of the effectiveness of psychological, cognitive, lifestyle and pharmacological interventions in Subjective Cognitive Decline (SCD).

Rather than solely focussing on cognitive outcome measures, this systematic review also evaluates the effectiveness of the interventions on psychological well-being and metacognition.

The studies included in the systematic review are generally of a low quality and this makes it difficult to draw firm conclusions.

For peer review only

INTRODUCTION

Subjective cognitive decline (SCD) describes self-reported acquired difficulties with cognitive functioning, in the presence of normal performance on objective measures of cognition and no impairment of daily functioning.[1] SCD has attracted alternative names, including subjective memory impairment (SMI) and subjective memory decline (SMD). The reported prevalence of SCD is variable ranging from 18%-55% of memory clinic attendees.[2-5] However, there is no evidence based consensus on best management for SCD and the priority of memory services is on treating patients with cognitive difficulties arising from established neurodegenerative disorders.[6]

The difficulty in treating patients with SCD stems from its aetiological heterogeneity. Broadly, two distinct groups of patients exist; those in whom SCD represents preclinical Alzheimer's disease (AD) and those in whom it does not. There is accumulating evidence for the former with Mitchell's meta-analysis[7] finding that older people with SCD were twice as likely to subsequently develop dementia. Indeed, patients presenting with SCD may already have subclinical and subtle cognitive decline suggesting an early manifestation of neurodegeneration.[8] Furthermore, there is a positive correlation between subjective cognitive complaints and amyloid deposition[9,10] and imaging studies have found brain changes indicative of AD in some patients with SCD.[11,12] However, SCD represents a heterogenous patient group and patients without preclinical AD are less likely to demonstrate these changes.

These findings have led to growing interest in SCD, especially in the context of prodromal AD, because targeted treatments at this earlier stage could have the potential to slow or even prevent progression to AD. However, this should not detract from the reality that the majority of patients with SCD do not have preclinical AD. A recent observational study found only 14% of patients with SCD progressed to mild cognitive impairment (MCI) or AD over a six year follow-up[13] whilst a systematic review[14] highlighted that SCD symptoms frequently remit. For this non pre-clinical AD group, functional psychiatric illness, personality traits, physical illness, psychosocial stress and the effects of alcohol and drug use are likely to be contributory factors.

Research into cerebrospinal fluid (CSF)[15] and neuroimaging biomarkers[9,10,16]suggest they hold promise for distinguishing between preclinical and non-preclinical AD in patients with SCD but they are not routinely used in clinical practice. Therefore, currently, for the vast majority of patients with SCD, it is not possible to make this important distinction in a timely manner and, often, only regular follow-up, over a number of years,[13,17] will reveal the underlying aetiology.

As well as identifying preclinical neurodegenerative dementias, there is an additional aspect to treating patients with SCD that must not be overlooked and this relates to the distressing symptoms that they experience. Self-perception of cognitive difficulties can lead to anger, increased stress and fear of dementia[18] while there is growing evidence that features of anxiety and depression are common in patients with SCD.[19] Therefore, patients with SCD, regardless of aetiology, would benefit from interventions that improve psychological well-being.

Given the lack of evidence-based treatments to offer patients with SCD in the clinic, alternative options need to be sought. Metacognition, an individual's insight into their own cognitive functioning,[20]may be of relevance as a therapeutic target because, by definition, SCD involves an individual's underestimation of their cognitive ability relative to objective testing. Metternich[21] demonstrated that this patient group had significantly poorer metacognitive abilities than controls. As an over-estimate of cognitive impairment may be related to difficulties in evaluating cognitive

For peer review only

performance (i.e. metacognitive difficulties), improving metacognitive ability may reduce the subjective cognitive impairment experienced by these patients.

Three systematic reviews evaluating interventions for SCD have been published.[18,22,23] Metternich and colleagues'[18] found that 'expectancy change', which included interventions such as cognitive restructuring or psychoeducation to change beliefs about cognition, was significantly more effective at improving metacognition than a non-active control intervention. A limitation of this systematic review was that nine of the 14 studies included involved participants who were healthy volunteers and did not necessarily have SCD. Cavenelli[22] reviewed six studies of cognitive interventions for SCD and found improvements in objective cognitive performance, often limited to the cognitive domain being trained. The most recent systematic review[23] on this topic, perhaps benefitting from being the first since the conceptualisation of the term SCD,[1] used a more rigorous inclusion criteria and found a small effect size for all non-pharmacological interventions (NPI).

Purpose of current review

There is no consensus on the best treatment for SCD. We acknowledge the importance, both clinically and in research, of distinguishing between preclinical and non-preclinical dementia types of SCD but are mindful that this is often difficult in the majority of cases in clinical practice. Therefore, this review is a pragmatic analysis of which treatment, if any, should be offered to patients presenting with SCD in clinical settings when CSF and neuroimaging biomarker investigations are not routinely available.

In order to establish the best current treatment evidence, we aimed to conduct a systematic review of all types of interventions (psychological, cognitive, lifestyle and pharmacological) and to evaluate their effects on psychological well-being, metacognition and objective cognitive performance. Psychological well-being was chosen as an outcome measure because psychological distress is common in SCD,[18,19]metacognitive ability because it may be inversely linked to subjective cognitive complaints[21] especially in non-preclinical dementia SCD, and objective cognitive performance as this has previously been identified as the main outcome in intervention studies in SCD[23] and may reflect the subtle impairment found in this patient group.

For peer review only

METHODS

Search Strategy and selection criteria

We searched PubMed, Web of Science, Cochrane Systematic Reviews Database, PsycINFO and CIANHL in August 2017. Search terms used were: "memory", "cognitive" or "forgetfulness" AND "subjective", "functional", "benign", "self-reported", "complaints", "complainer" or "healthy" AND "training", "intervention", "treatment", "therapy" or "medicine" (see online supplementary appendix 1 for further details on search strategy). We did not apply limits for language or date of publication. Furthermore, reference lists of included studies were manually scanned for additional relevant papers.

Inclusion and Exclusion Criteria

We included studies where participants, either all or a separately analysed subgroup, had SCD defined as being self-reported cognitive complaints, without objective evidence of deficits on cognitive testing and unaccounted for by medical or psychiatric causes.[1] The intervention delivered needed to fall within the domains of psychological, cognitive, lifestyle or pharmacological. During data extraction, three authors (RB, AB and JH) independently assigned the study intervention to one of these domains and when there were multiple components they judged this on the most predominant activity. Outcomes of interest included psychological well-being, metacognition and objective cognitive performance. Studies with active and non-active comparator groups were included. There was no age restrictions and only RCTs were included. Studies including patients with mild cognitive impairment (MCI) or dementia were excluded as were those including patients with significant psychiatric or physical co-morbidities.

Assessment of trial Quality

In order to assess the risk of bias, three authors (RB, AB and JH) independently rated study validity using criteria that were based on the Critical Appraisal Skills Programme (CASP; www.hello.nhs.uk/documents/CAT6-Randomised_Controlled_Trials.pdf) and have been used previously.[24,25]

Five criteria evaluating quality and bias were assessed and based on whether studies reported the following:

1. Randomisation of participants to treatment and control groups.
2. Blinding of participants and clinicians, where possible, to treatment allocation.
3. Use of 'intention to treat' analysis for incomplete outcome data.
4. Participant follow up and data collection equal in all groups.
5. An a priori power calculation based on at least one of our outcomes of interest.

Any disagreements between raters (RB, AB and JH) were resolved through discussion with a fourth author (RH). Each checklist item above scored 1 point, therefore each study could score between 0 and 5 for validity. As with previous work[25] studies which independently randomised participants to

For peer review only

treatment and control groups, used intention to treat analysis to manage incomplete outcome data and followed up patients in the same way (points 1, 3 and 4 above) were deemed to be high quality.

Data extraction

The studies' demographic details, participant characteristics, main findings, limitations and results in the form of means and standard deviation for each outcome measure were independently extracted by three authors (RB, AB and JH).

Statistical analysis

We compared intervention and control groups post-intervention using RevMan software. The programme uses Hedges adjusted g[26] to calculate a standardised mean difference (SMD) which is adjusted for small sample bias. Meta-analyses were performed where three or more studies investigated a comparable intervention and outcome using a random effects model. Heterogeneity was quantified using the I^2 statistic.

Where a study reported multiple outcome measures for one outcome domain (eg within psychological well-being) we calculated a composite measure to provide a single quantitative measure for meta-analysis.[27]

For example, if a study reported two relevant outcomes, expressed as two effect sizes (y_1 and y_2). The overall mean effect size for the composite measure will be:

$\bar{y} = ½ (y_1 + y_2)$

The variance of the composite effect size is calculated as follows:

$V_{\bar{y}} = ¼ (V_{Y1} + V_{y2} + 2r*\sqrt{V_{Y1}}*\sqrt{V_{y2}})$,

where r is the correlation coefficient describing to what extent y_1 and y_2 co-vary. In the absence of existing literature to identify a suitable correlation, we reported composite effect sizes calculated using a correlation of 0.5.

Further details of statistical methods are found in the supplementary material (see online supplementary appendix 1).

Patient and Public Involvement

There was no direct patient or public involvement in this review.

For peer review only

RESULTS

Description of studies

Sixty-three potentially relevant articles were retrieved through our initial search. Of these, 20 studies met inclusion criteria, however only 16 studies had sufficient outcome data to allow calculation of effect sizes for inclusion in meta-analyses (Figure 1). The characteristics and validity of included studies are described in the online supplementary material (online supplementary data file 2: Table DS1).

Of the twenty included studies, five were classified as psychological interventions; four as group psychological treatment[28-31] and one as mindfulness training.[32] Eleven studies were classified as cognitive or memory training,[28,33,34-42] two as lifestyle[35,43] and four as pharmacological[44-47] interventions. Three studies[31,33,46] examined two differing interventions from the same category while two studies investigated interventions from different categories.[28,35]

Participants were community dwelling in all of the studies although the source of recruitment varied; memory clinics in two studies,[30,42] community centres in three studies[28,37,38] and the remainder through more widespread advertisement.

Of the RCTs included in the meta-analysis, twelve had a non-active control[28-30,33,36,39,41,43-47] while four had an active control.[32,35,37,40] The studies included a range of outcomes and only five RCTs[29,30,35,37,44] clearly stated their primary outcome a priori. Fifteen RCTs[28,30,32,33,35-37,39-41,43-47] included in the meta-analysis included objective cognitive performance amongst their outcomes but only three of these used an assessment of global cognitive function.[36,37,40] Ten studies[28,30-34,38,39,41,43,46,47] reported metacognitive outcomes, of which three provided a global measure of metacognition.[31,33,41] Seven studies[28,30,32,33,36,39,44] reported psychological well-being outcomes. All of the RCTs reported outcome data within two weeks of the intervention ending and four studies[30],[39-41] collected further follow-up data up ranging from one to six months post-intervention.

Quality of studies

According to criteria described in the methods section, seven studies[29,30,35,41,43,44,47] included in the meta-analyses were rated as being of high quality. An a priori power calculation was only carried out in two studies[30,35] while the analysis of incomplete outcome data was adequately addressed in only eight studies.[29,30,35,41,43,44,47] Thirteen[29-31,33, ,35-37,39,41-47] out of 16 studies met criteria for adequate randomisation of participants and the same number of studies[29,30,32,33,35,36,39,41-47] ensured adequate blinding of participants and researchers. Publication bias was assessed using funnel plots for each outcome of interest (see online supplementary appendix 2; Figure DS1).

Group psychological interventions (four studies)

Four small studies[28-31] investigated group psychological interventions. Three[29-31] were assessed to be high quality. Their main focus included psychoeducation about cognitive ageing and the link between cognitive difficulties and stress or anxiety together with goal management, strategies to improve metacognition and relaxation training. One RCT[31] which showed a reduction in anxiety symptoms in the intervention group compared to the control group, could not be included in meta-

For peer review only

analyses due to insufficient baseline data. The meta-analyses for the three remaining studies showed a significant improvement in psychological well-being post-intervention (g=0.40, 95% CI 0.03-0.76, Z=2.12, P=0.03, total n=65, see figure 2).

When meta-analysed, group psychological interventions[28-30] conferred a positive effect on metacognition but this was not statistically significant (g=0.26, 95% CI -0.22-0.73, Z= 1.07, P=0.28, total n=65, see figure 3). Interestingly, Metternich and co-workers[30] found that their group based therapy did not improve memory self-efficacy (MSE) immediately post-intervention but there was a significant improvement in MSE at three months follow up (P=0.04).

Three of the studies[28,29,31] measured objective cognitive performance. Hoogenhout[29] did not find any significant differences in objective measures of memory (U=229, p=0.161, n=30) or executive functioning and speed (U=274, p=0.616, n=30). Similarly there was no effect on executive function, the only cognitive domain evaluated by Van Hooren et al.[31] In Pereira-Morales' study[28] the Integrated Psychostimulation Program (IPP), which contains cognitive training, conferred a significant improvement in a composite measure of objective cognitive performance (see figure 4).

Mindfulness (One study)

Smart et al[32] conducted a pilot RCT (N=15) to assess the feasibility of mindfulness in SCD. The control group received psychoeducation and both groups demonstrated an improvement in MSE together with a reduction in cognitive complaints.

Lifestyle interventions (two studies)

A high quality factorial design RCT[35] showed no benefit of aerobic exercise on objective cognitive performance compared to an active control (gentle exercise that did not increase heart rate) (g=0.04, CI -0.25-0.33, n=32, see figure 4). However, there was significant improvement in both groups.

Small's[43] small but high quality RCT found that an intervention to promote healthy lifestyle strategies did not improve metacognition (g=-0.11, CI -0.80-0.58, n=8, see figure 3). There was an improvement, albeit not reaching statistical significance, in a composite measure of verbal learning and memory and verbal fluency (g=0.42, CI -0.42-1.26, n=8, see figure 4). The small sample size and lack of monitoring of the compliance of participants prevents any firm conclusions being drawn.

Cognitive training interventions (11 studies)

Thirteen cognitive or memory training interventions across 11 RCTs,[28,33-42] were evaluated. Three studies[34,38,42] provided insufficient data to be included in meta-analyses. Only two studies[35,41] were rated as high quality. For objective cognitive performance, significant pooled effect sizes were found (g=0.13, CI 0.01-0.25, Z=2.13, P=0.03, total n=311, see figure 4) with no heterogeneity (I^2=0%). Three studies[35,37,40] had active control groups in which participants received forms of cognitive stimulation and when analysed separately, their pooled effect size was equivocal (g=0.02 CI -0.19-0.22, Z=0.19, P=0.85, total n=132, see figure 5). Conversely, for the remaining studies which used non-active controls, the pooled effect size was significant (g=0.18, CI 0.04-0.33, Z=2.48, P=0.01, total n=179, see figure 5).

Three studies[36,37,40] used global cognitive performance outcome measures and when meta-analysed there was no discernible improvement post intervention (g=0.06, CI=-0.19-0.31, Z=0.45, P=0.66, total n=114, see supplementary appendix 2: Figure DS2).

For peer review only

Five studies[28,33,36,37,39] reported psychological well-being. Each of the studies demonstrated an improvement in psychological well-being but individually none were statistically significant. However, when meta-analysed there was a small beneficial effect on psychological well-being (g=0.25, CI= 0.05-0.46, Z=2.40, P=0.02, total n=169, see figure 2) with no heterogeneity (I^2=0). Three studies[33,39,42] reported metacognitive outcomes and pooled together there was no significant effect (g=0.06, CI -0.12-0.24, Z=0.66, P=0.51, total n=165, see figure 3).

Pharmacological interventions (4 studies)

Two studies investigated the effectiveness of Gingko biloba. Brautigam's low quality but relatively large trial[46] included two different dosing regimens, namely high and low dose Gingko extract for 24 weeks. They led to conflicting results with high dose Gingko causing a statistically significant worsening in metacognition while low dose Gingko produced the opposite effect although statistically insignificant.

In Beck and colleagues' trial,[44] a 240mg preparation of Gingko Biloba was administered to the intervention group daily for eight weeks. Both psychological well-being and objective cognitive performance improved but neither was statistically significant.

One small, low quality trial by Boespflug et al[45] found that 2.4g fish oil daily had no effect on working memory.

Zhu and colleagues[47] evaluated the effectiveness of BrainPower Advanced, a dietary supplement, taken twice daily for 12 weeks. The trial was of a poor quality with marked baseline differences in severity of cognitive complaints between groups. Analysing the primary data, there was no significant difference in metacognitive ability (g=0.07, CI -0.35-0.31, n=47, see figure 3) or objective cognitive performance (g=0.11, CI -0.28-0.50, n=47, see figure 4) between the groups.

For peer review only

DISCUSSION

Main Findings

The most obvious finding from our review is the lack of good quality evidence in this field. However, given that this is an emerging field, we attempted to be as inclusive as possible and included low quality studies in our meta-analyses provided they did not have an inadequate or unclear rating in all five areas of bias that we assessed (see methods).

There was a statistically significant improvement in psychological well-being for interventions which incorporated psychoeducation and were delivered in group settings, although the effect size was only moderate (g=0.40).[48] The studies, two of which were of high quality, are limited by small sample sizes and one of the interventions[28] included cognitive training in addition to a psychological approach. Overall, however, this could be a promising intervention that can be feasibly implemented into clinical practice.

We found that cognitive interventions conferred a small but statistically significant effect (g=0.13) on cognitive outcomes which is in keeping with Smart's earlier review.[23] However, the clinical relevance of such a small improvement is easy to question. The apparent clinical utility of cognitive interventions in SCD is further undermined by our finding that meta-analysis of the three studies which used global cognitive performance outcome measures found no overall benefit. Our use of composite measures provided us with a single effect size for cognitive performance in each study but we are mindful that this merely reflects performance across the cognitive domains assessed and is not a surrogate for global cognition. Smart[23] suggested that the small improvements in cognition are important because they demonstrate that cognitive enhancement and compensation is possible in this patient group. While this may be of relevance when considering the small but significant minority of persons in whom SCD represents pre-clinical AD, this finding has less bearing on the remaining majority who, by definition, have normal objective cognitive performance.

We found that cognitive training conferred a small but significant benefit on psychological well-being. The effects of cognitive training on psychological well-being have been poorly researched[49] and there is sometimes an unproven assumption that cognitive training enhances a patient's overall well-being by preserving cognitive function.[50] This would not explain our findings given patients were evaluated within a short time frame from baseline. The larger effect on psychological well-being than on objective cognitive performance measures may reflect a significant psychogenic aetiology in many cases of SCD. However, it is important to note that the evidence is of a poor quality and this may represent a chance finding given that psychological well-being was not a primary outcome for any of the cognitive training intervention studies.

Metacognitive deficits are relevant in subjective cognitive disorders.[21] Psychoeducation delivered in group settings did not lead to a significant improvement in metacognitive ability immediately after the intervention but this finding was influenced by Metternich and colleagues'[30] study which found less improvement in the intervention group compared to the control (g=-0.30, CI= -1.01-0.41). However, their intervention led to a significant improvement in metacognitive ability when assessed three months post-treatment leading them to postulate that a period of time is needed for cognitive restructuring and the implementation of strategies into everyday life. Our findings suggest that further work is required to explore the role of group psychoeducation in improving metacognition

For peer review only - http://bmjopen.bmj.com/site/about/guidelines.xhtml

and longer lengths of follow up are indicated. A limitation to the literature investigating metacognitive outcomes is the broad range of subjective measures in use.

There is only limited evidence available with regards to lifestyle interventions for SCD. Exercise was evaluated in one, high-quality RCT. The intervention group performed aerobic exercise while the control group performed gentle exercise designed not to increase heart rate. Both groups showed significant improvements in objective cognitive performance but there was no difference between the groups. This may suggest that exercise, regardless of intensity, could be beneficial in SCD and further RCTs with a non-active control group are indicated.

Five studies have investigated alternative medicines and medicinal supplements in SCD. Overall, the evidence for their efficacy is not encouraging. One study[44] found that 240mg daily of Gingko Biloba for eight weeks led to non-significant improvements in psychological well-being and cognitive performance. Brautigam and colleagues[46] used two alternative doses of Gingko Biloba and their results are conflicting. Higher doses led to significantly worse metacognition compared to control and lower doses led to statistically insignificant improvements. Fish oil[45] did not improve objective cognitive performance while a dietary supplement[47] did not improve either metacognition or objective cognitive performance.

In summary, psychoeducation delivered in group settings appears to be effective at improving psychological well-being, which is a relevant and important outcome, in SCD.[18,19] However, individually none of the three studies demonstrated statistically significant improvements which was largely due to their small sample sizes. A large RCT is urgently needed to extrapolate our findings for this clinically practical intervention which is highly promising. Metacognition, an individual's insight into their own cognitive functioning, is a potential area of research in SCD because deficits in this area may contribute to the condition, especially the non-preclinical dementia type. Specific interventions to improve metacognitive performance as well as to objectively quantify it are emerging[20] and these may be of relevance in SCD. Cognitive interventions have a modest effect in improving objective cognitive performance and such small improvements are unlikely to be of clinical significance for the vast majority of patients with SCD who are not at risk for developing dementia. There is limited research considering lifestyle interventions in SCD and only unconvincing and poor quality evidence for pharmacological interventions in SCD.

Limitations

There is a high variability in measurement instruments used to define SCD in studies[51] which limits reviews such as our own. Furthermore, SCD is a construct which includes a heterogenous group of patients that could be broadly divided into two distinct patient groups; those with and without preclinical dementia. All of the studies in our review included both groups of SCD patients together and this limits our ability to make inferences about targeted interventions for each group. Future work should investigate interventions for each group of patients separately if possible.

The poor quality of studies in this review due to a lack of clearly defined primary outcomes and small sample sizes has been highlighted previously and limits the confidence with which we can draw conclusions. Funnel plots assessing publication bias (see online supplementary appendix 2; Figure DS1) appeared asymmetrical. Small sample sizes and heterogeneous interventions may be contributory factors but we are mindful that when the number of studies in the meta-analysis is small as is the case in this review, such asymmetry may arise by chance rather than reflect true publication bias.[52]

For peer review only

The interventions were heterogenous. As well as differing formats and structures of interventions which we attempted to group into broad categories, there were differences in lengths of intervention, monitoring of compliance and the motivation of participants which are more difficult to quantify. Often interventions had multiple components and we had to determine which domain was the most prominent. This may mean the most effective component of the intervention was not appropriately captured.

Similarly, the studies included in the meta-analysis contained varied control groups. In at least two[32,40] of the four studies using an active control group, the control group received treatment which may have been effective in its own right thereby masking the possible effectiveness of the intervention. The other studies used non-active control groups but there is likely to have been much variability in what treatment as usual constituted.

The goal of research into interventions must be for them to be translational into clinical practice. Unfortunately, only one study[30] included in the meta-analyses recruited patients from a healthcare setting whilst the remainder relied on convenience sampling of volunteers with subjective cognitive complaints from the community. This may limit how translatable our findings are to clinical populations.

Implications

This review demonstrates the need for higher quality studies in SCD research with a distinction made between SCD in pre-clinical AD and otherwise. The development of a conceptual framework for research in SCD[1] should aid this. This review would support high quality RCTs of a group psychological intervention targeting psychological well-being and metacognition in patients with SCD. Follow-up data at least three months post-intervention should be collected. Metacognitive ability may be an important therapeutic target in SCD and interventions to directly improve this along with objective measurement tools are required.

For peer review only

Contributors

All authors made a substantial contribution to this work. RB, AB, JH and RH all contributed to the conception and design of the review. RB read and screened abstracts and titles of potentially relevant studies. RB, AB and JH read the retained papers and were responsible for extracting data and rating their quality independently. RB drafted the paper with all the authors critically reviewing it and suggesting amendments prior to submission. All the authors had access to all the data in the study and can take responsibility for the integrity of the reported findings.

Funding

This research received no specific grant from any funding body. RB is supported by a NIHR Academic Clinical Fellowship. JH and RH are supported by the NIHR UCLH BRC.

Competing Interests

None declared.

Data sharing statement

Details of excluded papers are available from the first author on request.

For peer review only

Figure legends

Figure 1. Details of search strategy. RCT, Randomised control trial.

Figure 2. Forest plot comparing interventions (all types) vs control (all types) for patients with subjective cognitive decline. Outcome: Psychological well-being (at the end of intervention).

Figure 3. Forest plot comparing interventions (all types) vs control (all types) for patients with subjective cognitive decline. Outcome: Metacognitive ability (at the end of intervention).

Figure 4. Forest plot comparing interventions (all types) vs control (all types) for patients with subjective cognitive decline. Outcome: Objective cognitive performance (at the end of intervention).

Figure 5. Forest plot comparing cognitive interventions vs non-active controls and active controls in separate analyses for patients with subjective cognitive decline. Outcome: Objective cognitive performance (at the end of intervention).

Figure DS1. Funnel plot as indicator of publication bias. All studies for a) psychological well-being outcomes; b) metacognitive ability outcomes and c) objective cognitive performance outcomes.

Figure DS2. Forest plot comparing cognitive interventions vs control (all types). Outcome: Objective global cognitive performance (at the end of intervention).

For peer review only

REFERENCES

1. Jessen F, Amariglio RE, van BM, et al. A conceptual framework for research on subjective cognitive decline in preclinical Alzheimer's disease. Alzheimers Dement 2014;10(6):844-852.

2. Elfgren C, Gustafson L, Vestberg S, et al. Subjective memory complaints, neuropsychological performance and psychiatric variables in memory clinic attendees: a 3-year follow-up study. Arch Gerontol Geriatr 2010;51(3):e110-e114.

3. Alladi S, Arnold R, Mitchell J, et al. Mild cognitive impairment: applicability of research criteria in a memory clinic and characterization of cognitive profile. Psychol Med 2006;36(4):507-515.

4. Wahlund LO, Pihlstrand E, Jonhagen ME. Mild cognitive impairment: experience from a memory clinic. Acta Neurol Scand Suppl 2003;179:21-24.

5. Vraamark ET, Stokholm J, Hogh P, et al. Diagnostic profile of young and middle-aged memory clinic patients. Neurology 2002;59(8):1259-1262.

6. Stone J, Pal S, Blackburn D, et al. Functional (Psychogenic) Cognitive Disorders: A Perspective from the Neurology Clinic. J Alzheimers Dis 2015;48 Suppl 1:S5-S17.

7. Mitchell AJ, Beaumont H, Ferguson D, Yadegarfar M, Stubbs B. Risk of dementia and mild cognitive impairment in older people with subjective memory complaints: meta-analysis. Acta Psychiatr Scand 2014;130(6):439-451.

8. Koppara A, Wagner M, Lange C, Ernst A, Wiese B, Konig HH et al. Cognitive performance before and after the onset of subjective cognitive decline in old age. Alzheimers Dement (Amst) 2015; 1(2):194-205.

9. Amariglio RE, Becker JA, Carmasin J, et al. Subjective cognitive complaints and amyloid burden in cognitively normal older individuals. Neuropsychologia 2012;50(12):2880-2886.

For peer review only

10. Perrotin A, Mormino EC, Madison CM, et al. Subjective cognition and amyloid deposition imaging: a Pittsburgh Compound B positron emission tomography study in normal elderly individuals. Arch Neurol 2012;69(2):223-229.

11. Perrotin A, de Flores R, Lamberton F, et al. Hippocampal Subfield Volumetry and 3D Surface Mapping in Subjective Cognitive Decline. J Alzheimers Dis 2015;48 Suppl 1:S141-S150.

12. Peter J, Scheef L, Abdulkadir A, Boecker H, Heneka M, Wagner M et al. Gray matter atrophy pattern in elderly with subjective memory impairment. Alzheimers Dement 2014;10(1):99-108.

13. Hessen E, Eckerstrom M, Nordlund A, Selseth A, I, Stalhammar J, Bjerke M et al. Subjective Cognitive Impairment Is a Predominantly Benign Condition in Memory Clinic Patients Followed for 6 Years: The Gothenburg-Oslo MCI Study. Dement Geriatr Cogn Dis Extra 2017;7(1):1-14.

14. Mendonca MD, Alves L, Bugalho P. From Subjective Cognitive Complaints to Dementia: Who is at Risk?: A Systematic Review. Am J Alzheimers Dis Other Demen 2016;31(2):105-114.

15. Wolfsgruber S, Polcher A, Koppara A, Kleineidam L, Frolich L, Peters O et al. Cerebrospinal Fluid Biomarkers and Clinical Progression in Patients with Subjective Cognitive Decline and Mild Cognitive Impairment. J Alzheimers Dis 2017; 58(3):939-950.

16. Vogel JW, Varga DM, La JR, Marks SM, Schwimmer HD, Landau SM et al. Subjective cognitive decline and beta-amyloid burden predict cognitive change in healthy elderly. Neurology 2017; 89(19):2002-2009.

17. Eckerstrom M, Gothlin M, Rolstad S, Hessen E, Eckerstrom C, Nordlund A et al. Longitudinal evaluation of criteria for subjective cognitive decline and preclinical Alzheimer's disease in a memory clinic sample. Alzheimers Dement (Amst) 2017; 8:96-107

18. Metternich B, Kosch D, Kriston L, et al. The effects of nonpharmacological interventions on subjective memory complaints: a systematic review and meta-analysis. Psychother Psychosom 2010;79(1):6-19.

19. Molinuevo JL, Rabin LA, Amariglio R, Buckley R, Dubois B, Ellis KA et al. Implementation of subjective cognitive decline criteria in research studies. Alzheimers Dement 2017; 13(3):296-311.

For peer review only

20. Fleming SM, Dolan RJ. The neural basis of metacognitive ability. Philos Trans R Soc Lond B Biol Sci 2012;367(1594):1338-1349.

21. Metternich B, Schmidtke K, Hull M. How are memory complaints in functional memory disorder related to measures of affect, metamemory and cognition? J Psychosom Res 2009;66(5):435-444.

22. Canevelli M, Adali N, Tainturier C, et al. Cognitive interventions targeting subjective cognitive complaints. Am J Alzheimers Dis Other Demen 2013;28(6):560-567.

23. Smart CM, Karr JE, Areshenkoff CN, Rabin LA, Hudon C, Gates N et al. Non-Pharmacologic Interventions for Older Adults with Subjective Cognitive Decline: Systematic Review, Meta-Analysis, and Preliminary Recommendations. Neuropsychol Rev 2017;27(3):245-257.

24. Cooper C, Li R, Lyketsos C, Livingston G. Treatment for mild cognitive impairment: systematic review. Br J Psychiatry 2013;203(3):255-264

25. Rapaport P, Livingston G, Murray Jet al. Systematic review of the effective components of psychosocial interventions delivered by care home staff to people with dementia. BMJ Open 2017;7(2):e014177.

26. Hedges LV, Olkin I. Statistical methods for meta-analysis. New York, Academic Press 1985.

27. Borenstein M, Hedges LV, Higgins JPT, et al. Introduction to Meta-Analysis. Chichester, John Wiley & Sons, Ltd 2009.

28. Pereira-Morales AJ, Cruz-Salinas AF, Aponte J, et al. Efficacy of a computer-based cognitive training program in older people with subjective memory complaints: a randomized study. Int J Neurosci 2018;128(1):1-9.

29. Hoogenhout EM, de Groot RH, van der Elst W, et al. Effects of a comprehensive educational group intervention in older women with cognitive complaints: a randomized controlled trial. Aging Ment Health 2012;16(2):135-144.

30. Metternich B, Schmidtke K, Harter M, et al. [Development and evaluation of a group therapy for functional memory and attention disorder]. Psychother Psychosom Med Psychol 2010; 60(6):202-210.

For peer review only

31. van Hooren SA, Valentijn SA, Bosma H, et al. Effect of a structured course involving goal management training in older adults: A randomised controlled trial. Patient Educ Couns 2007;65(2):205-213.

32. Smart CM, Segalowitz SJ, Mulligan BP, et al. Mindfulness Training for Older Adults with Subjective Cognitive Decline: Results from a Pilot Randomized Controlled Trial. J Alzheimers Dis 2016;52(2):757-774.

33. Oh SJ, Seo S, Lee JH, et al. Effects of smartphone-based memory training for older adults with subjective memory complaints: a randomized controlled trial. Aging Ment Health 2017;1-9.

34. Andrewes DG, Kinsella G, Murphy M. Using a memory handbook to improve everyday memory in community-dwelling older adults with memory complaints. Exp Aging Res 1996;22(3):305-322.

35. Barnes DE, Santos-Modesitt W, Poelke G, et al. The Mental Activity and eXercise (MAX) trial: a randomized controlled trial to enhance cognitive function in older adults. JAMA Intern Med 2013;173(9):797-804.

36. Cohen-Mansfield J, Cohen R, Buettner L, et al. Interventions for older persons reporting memory difficulties: a randomized controlled pilot study. Int J Geriatr Psychiatry 2015;30(5):478-486.

37. Kwok TC, Bai X, Li JC, et al. Effectiveness of cognitive training in Chinese older people with subjective cognitive complaints: a randomized placebo-controlled trial. Int J Geriatr Psychiatry 2013;28(2):208-215.

38. Pike KE, Ong B, Clare L, et al. Face-name memory training in subjective memory decline: how does office-based training translate to everyday situations? Neuropsychol Dev Cogn B Aging Neuropsychol Cogn 2017;1-29.

39. Scogin F, Storandt M, Lott L. Memory-skills training, memory complaints, and depression in older adults. J Gerontol 1985;40(5):562-568.

40. Tsai AY, Yang MJ, Lan CF, et al. Evaluation of effect of cognitive intervention programs for the community-dwelling elderly with subjective memory complaints. Int J Geriatr Psychiatry 2008;23(11):1172-1174.

For peer review only

41. Valentijn SA, van Hooren SA, Bosma H, et al. The effect of two types of memory training on subjective and objective memory performance in healthy individuals aged 55 years and older: a randomized controlled trial. Patient Educ Couns 2005;57(1):106-114.

42. Youn JH, Lee JY, Kim S, et al. Multistrategic memory training with the metamemory concept in healthy older adults. Psychiatry Investig 2011;8(4):354-361.

43. Small GW, Silverman DH, Siddarth P, et al. Effects of a 14-day healthy longevity lifestyle program on cognition and brain function. Am J Geriatr Psychiatry 2006;14(6):538-545.

44. Beck SM, Ruge H, Schindler C, et al. Effects of Ginkgo biloba extract EGb 761(R) on cognitive control functions, mental activity of the prefrontal cortex and stress reactivity in elderly adults with subjective memory impairment - a randomized double-blind placebo-controlled trial. Hum Psychopharmacol 2016;31(3):227-242.

45. Boespflug EL, McNamara RK, Eliassen JC, et al. Fish Oil Supplementation Increases Event-Related Posterior Cingulate Activation in Older Adults with Subjective Memory Impairment. J Nutr Health Aging 2016;20(2):161-169.

46. Brautigam MR, Blommaert FA, Verleye G, et al. Treatment of age-related memory complaints with Ginkgo biloba extract: a randomized double blind placebo-controlled study. Phytomedicine 1998;5(6):425-434.

47. Zhu J, Shi R, Chen S, et al. The Relieving Effects of BrainPower Advanced, a Dietary Supplement, in Older Adults with Subjective Memory Complaints: A Randomized, Double-Blind, Placebo-Controlled Trial. Evid Based Complement Alternat Med 2016:7898093.

48. Sullivan GM, Feinn R. Using Effect Size—or Why the P Value Is Not Enough. J Grad Med Educ 2012;4(3):279-282.

49. Bures V, Cech P, Mikulecka J, et al. The effect of cognitive training on the subjective perception of well-being in older adults. PeerJ 2016;4:e2785.

50. Woods B, Thorgrimsen L, Spector A, et al. Improved quality of life and cognitive stimulation therapy in dementia. Aging Ment Health 2006;10(3):219-226.

For peer review only

51. Rabin LA, Smart CM, Crane PK, Amariglio RE, Berman LM, Boada M et al. Subjective Cognitive Decline in Older Adults: An Overview of Self-Report Measures Used Across 19 International Research Studies. J Alzheimers Dis 2015; 48 Suppl 1:S63-S86.

52. Higgins JP, Green S (editors). Cochrane Handbook for Systematic Reviews of Interventions Version 5.1.0 [updated March 2011]. The Cochrane Collaboration 2011. Available from www.cochrane-handbook.org.

For peer review only

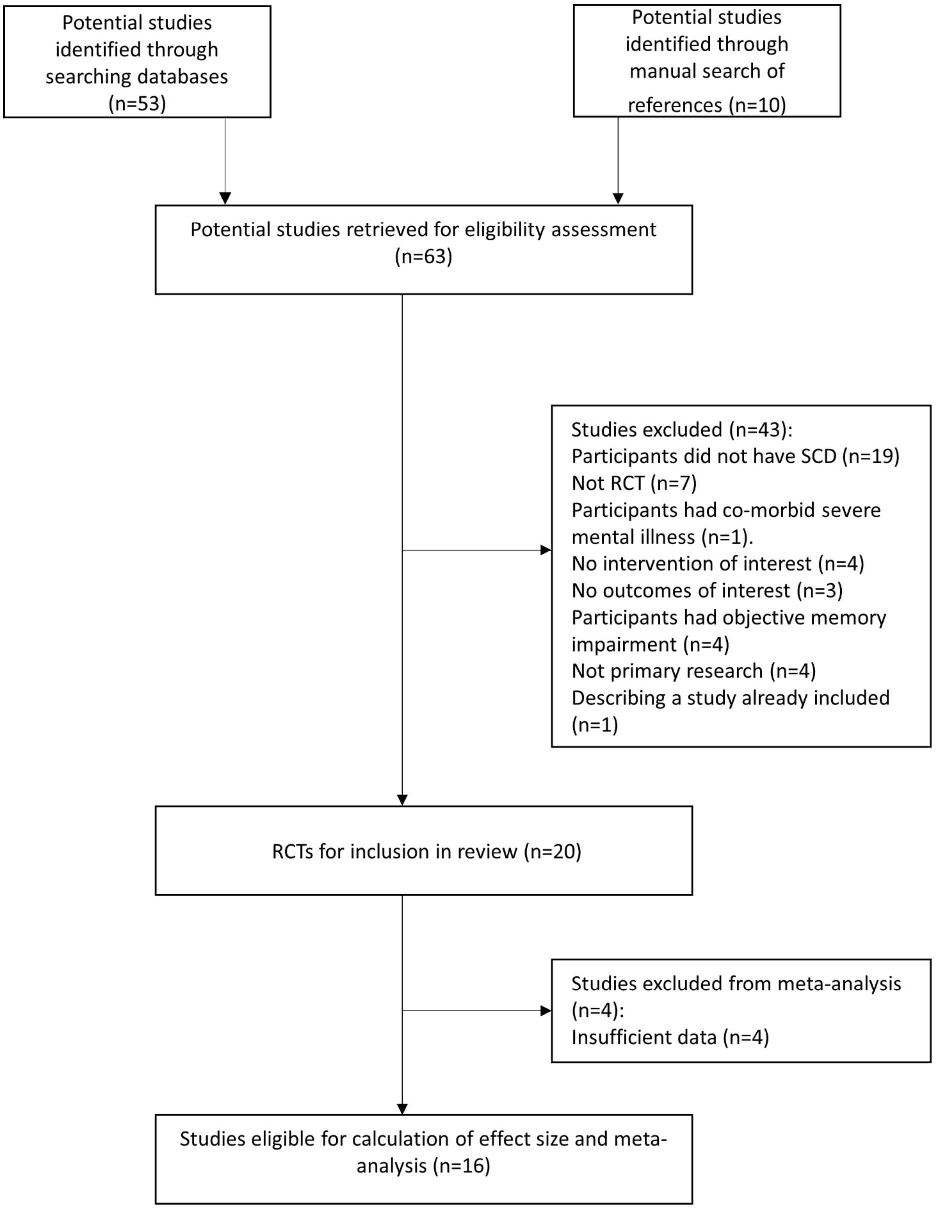

Figure 1. Details of search strategy. RCT, Randomised control trial.

175x227mm (300 x 300 DPI)

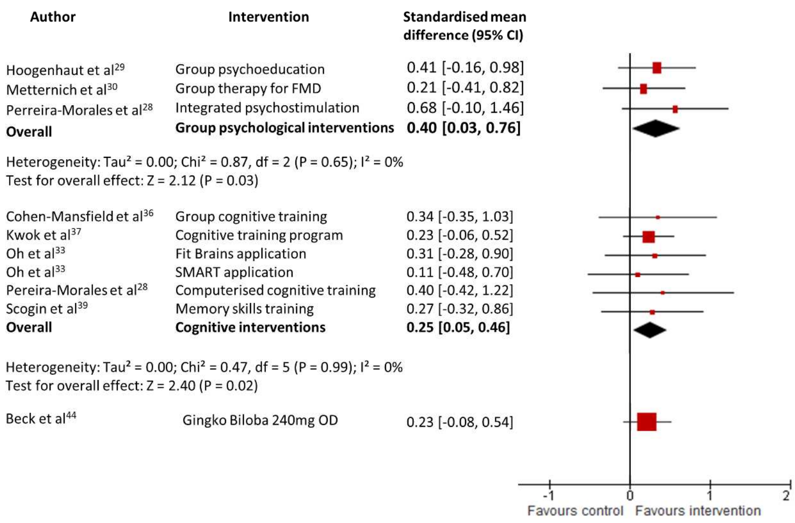

Figure 2. Forest plot comparing interventions (all types) vs control (all types) for patients with subjective cognitive decline. Outcome: Psychological well-being (at the end of intervention).

190x275mm (300 x 300 DPI)

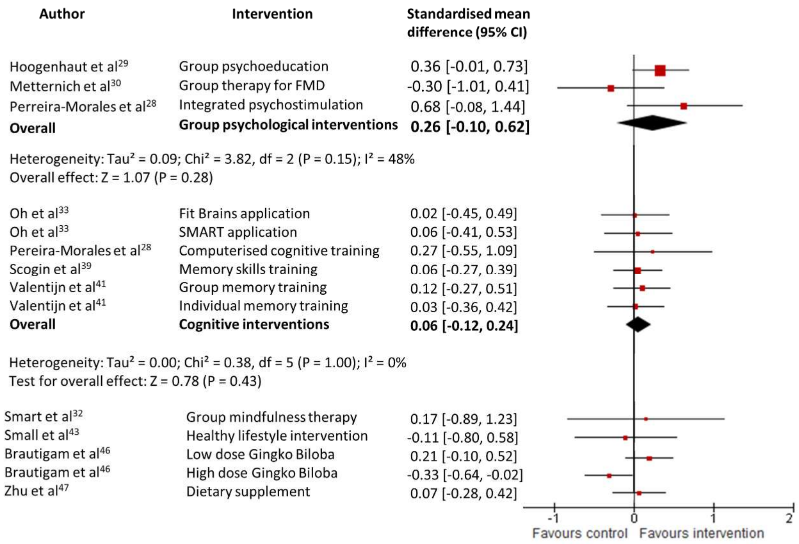

Figure 3. Forest plot comparing interventions (all types) vs control (all types) for patients with subjective cognitive decline. Outcome: Metacognitive ability (at the end of intervention).

190x275mm (300 x 300 DPI)

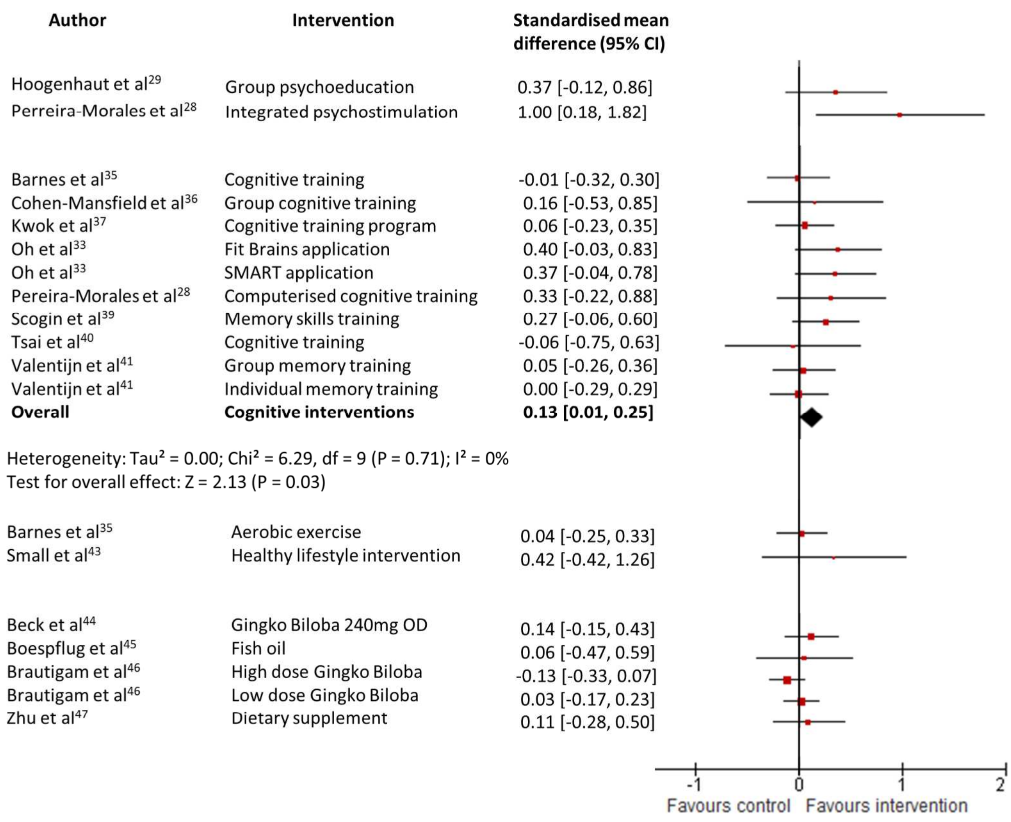

Figure 4. Forest plot comparing interventions (all types) vs control (all types) for patients with subjective cognitive decline. Outcome: Objective cognitive performance (at the end of intervention).

181x145mm (300 x 300 DPI)

For peer review only - http://bmjopen.bmj.com/site/about/guidelines.xhtml

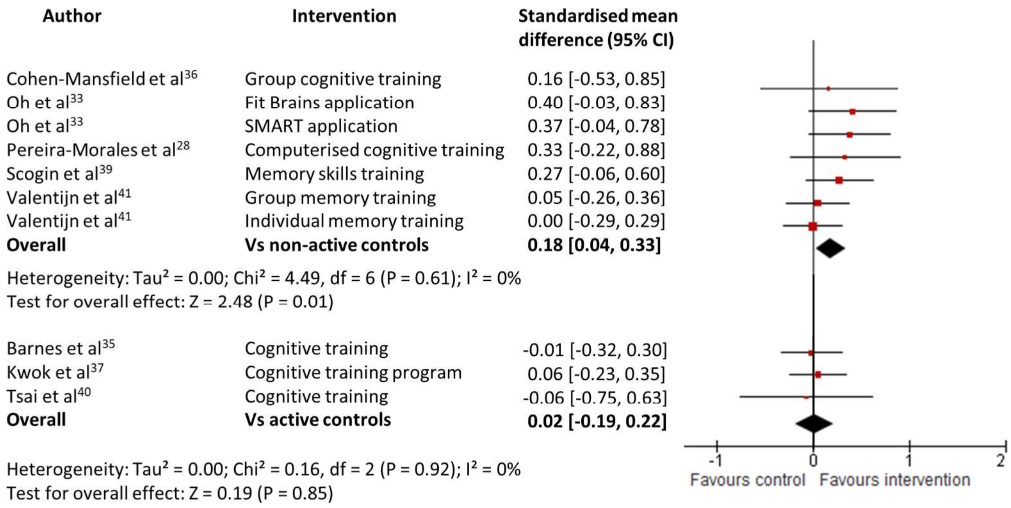

Figure 5. Forest plot comparing cognitive interventions vs non-active controls and active controls in separate analyses for patients with subjective cognitive decline. Outcome: Objective cognitive performance (at the end of intervention).

177x87mm (300 x 300 DPI)

For peer review only - http://bmjopen.bmj.com/site/about/guidelines.xhtml

Supplementary Appendix 1

Search Strategy (used in PubMed)

(“intervention” OR “treatment” OR “rehabilitation” OR “cognitive therapy” OR “cognitive training” OR “memory training” OR “computerised” OR “exercise” OR “cognitive behavioural” OR “cognitive behavioural” OR “metacognit*” OR “memory self- efficacy” OR “metamemory” OR “goal management” OR “expectancy change” OR “psychological” OR “group” OR “cognitive” OR “mindfulness” OR “Mediterranean diet” OR “mindfulness” OR “acupuncture” OR “meditation” OR “lifestyle” OR “diet” OR “gingko” OR “complementary” OR “vitamin*” OR “supplement*” OR “acetylcholinesterase inhibitor” OR “donepezil” OR “memantine” OR “galantamine” OR “rivastigmine”)

AND (“randomised controlled trial” OR “controlled trial” OR “intervention trial” OR “intervention study” OR “experimental study” OR “crossover trial” OR “RCT” OR “parallel”)

AND (“subjective cognitive decline” OR “subjective memory complain* OR “subjective cognitive concern” OR “subjective cognitive complain* OR “SCD” OR “SMC” OR “SCC” OR “preclinical dementia” OR “preclinical Alzheimer” OR “functional” OR “benign” OR “healthy”)

Statistical methods

Effect size calculation

Effect sizes were calculated using RevMan software. Standardised mean differences were calculated using Hedges’ adjusted g.

Pre-intervention standard deviations were used as these are most likely to be comparable across studies and therefore provide the most accurate estimate of effect size.[53]

The Hedges’ adjusted g formula[26] used in RevMan is as follows:

$$g= [M_{post\ intervention} – M_{post\ control}/SD_{pre\text{-}pooled}]*[1- 3/(4N-9)]$$

Where $N= n_{intervention\ group} + n_{control\ group}$

and

$$SD_{pre\text{-}pooled} = \surd\ [((n_{intervention\text{-}1})\ SD_{pre\ intervention}^2 + (n_{control\text{-}1})\ SD_{pre\ control}^2)/\ N\text{-}2]$$

Meta-analyses

Meta-analyses were performed using RevMan software. A random effects method as described by DeSirmonian and laird[54] was used. This adjusts standard errors of the effect sizes in each study to account for the heterogeneity for intervention effects observed between different studies.

The pooled effect size of each meta-analysis was calculated by attributing a weight to the average effect size in each study according to sample size. The z statistic was used to evaluate whether the pooled effect size was significantly different to no effect.

Heterogeneity was quantified using the I^s statistic.

Composite measure calculation

For peer review only - http://bmjopen.bmj.com/site/about/guidelines.xhtml

Composite scores were calculated where a study reported multiple outcomes falling within a particular outcome domain (e.g. objective cognitive performance). This approach was pragmatic in allowing one score to represent each intervention in the meta-analysis regardless of the number of outcomes reported.[27] The variance of the sum of variables was calculated as described below.

For example, if a study reported two relevant outcomes, expressed as two effect sizes (y_1 and y_2). The overall mean effect size for the composite measure will be:

$\bar{y} = \frac{1}{2}(y_1 + y_2)$

The variance of the composite effect size is calculated as follows:

$V_{\bar{y}} = \frac{1}{4}(V_{Y1} + V_{y2} + 2r * \sqrt{V_{Y1}} * \sqrt{V_{y2}})$,

where r is the correlation coefficient describing to what extent y_1 and y_2 co-vary.

If the correlation is set at 0, the outcomes are essentially treated as independent of each other and if the correlation is set at 1, the variance is an average of each outcome's variance. The former will lead to an underestimation of the variance and overestimation of precision while the latter will have the opposite effect. Consequently, in the absence of existing literature to identify a suitable correlation, we reported composite effect sizes calculated using a correlation of 0.5.

For peer review only

Supplementary appendix 2

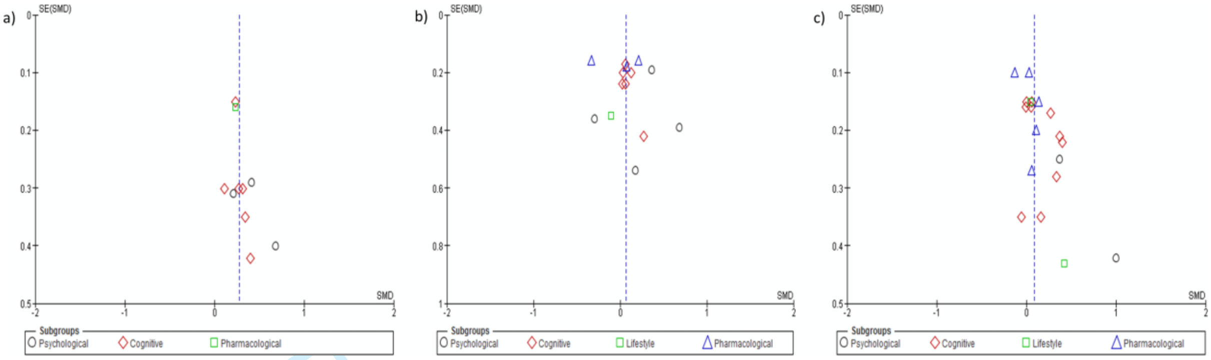

Figure DS1. Funnel plot as indicator of publication bias. All studies for a) psychological well-being outcomes; b) metacognitive ability outcomes and c) objective cognitive performance outcomes.

Author	Intervention	Standardised mean difference (95% CI)
Cohen-Mansfield et al[36]	Group cognitive training	0.16[-0.53, 0.85]
Kwok et al[37]	Cognitive training program	0.06 [-0.23, 0.35]
Tsai et al[40]	Cognitive training	-0.06 [-0.75, 0.63]
**Overall**	**Global cognitive performance**	0.06 [-0.19, 0.31]

Heterogeneity: $Tau^2 = 0.00$; $Chi^2 = 0.20$, df = 2 (P = 0.91); $I^2 = 0\%$
Test for overall effect: Z = 0.45 (P = 0.66)

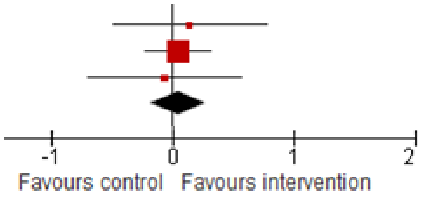

Fig. DS2 Forest plot comparing cognitive interventions vs control (all types). Outcome: Objective global cognitive performance (at the end of intervention).

Supplementary References

26. Hedges LV, Olkin I. Statistical methods for meta-analysis. New York, Academic Press 1985.

27. Borenstein M, Hedges LV, Higgins JPT, et al. Introduction to Meta-Analysis. Chichester, John Wiley & Sons, Ltd 2009.

53. Morris S. Estimating Effect Sizes From Pretest-Posttest-Control Group Designs. Organ. Res. Meth 2008;11(2):364-386.

54. DerSimonian R, Laird N. Meta-analysis in clinical trials. Control Clin Trials 1986; 7(3):177-188

For peer review only

Table DS1. Characteristics and validity of included studies

Study	Recruitment Setting	Inclusion criteria	Treatment group: Treatment	Treatment group: n	Control group: Treatment	Control group: n	Outcomes	Key findings: treatment vs control group, measured at end of intervention unless stated	Validity (answer to questions 1-5 in methods): 1	2	3	4	5
*Andrewes et al	Volunteers in the community	SMC; MDRS score > 123; Age 60-70 years	Memory training- participants provided with a handbook containing information and practice exercises to aid memory.<br><br>4 weeks	20	Non-active control- pamphlets providing information about three mnemonics.<br><br>4 weeks	20	Memory strategy knowledge questionnaire, Memory Strategy Use Questionnaire, memory diary.<br><br>Face-Name test, Prospective memory,	No differences in prospective memory tasks.<br><br>Significant improvement in face-name recall in memory training group.<br><br>No difference in cognitive measures at 4-months follow up.	y	y	y	y	n
Barnes et al	Volunteers in the community	SMC; Age >65 years	Mental activity performed on computers to enhance auditory and visual processing.<br><br>60 mins, 3 days a week for 12 weeks.	32	Active control- watched DVDs of educational lectures, on art, history and science.<br><br>60 mins, 3 days a week for 12 weeks	31	RAVLT, EFT, DSST, TMT-A, TMT-B, UFOV, Verbal fluency	Visuospatial function improved significantly for treatment group; improved global cognitive functioning in both groups but no significant inter-group differences.	y	y	y	y	y

For peer review only - http://bmjopen.bmj.com/site/about/guidelines.xhtml

Barnes et al	Volunteers in the community	SMC; Age>65 years	Aerobic exercise aiming to increase heart rates to 60-75% of participants' maximum.<br><br>60 mins, 3 days a week for 12 weeks	32	Active control-without aerobic exercise portion. Aim was not to increase resting heart rate-<br><br>60 mins, 3 days a week for 12 weeks	31	RAVLT, EFT, DSST, TMT-A, TMT-B, UFOV, Verbal fluency	Improved global cognitive functioning in both groups but no significant inter-group differences.	y	y	y	y	y
Beck et al	Volunteers in the community	SMI as diagnosed by the PRMQ.	Gingko Biloba 240mg OD<br><br>8 weeks	43	Placebo<br><br>8 weeks	32	Task set shifting, delayed response task, prospective memory task, Go-NoGo task,<br><br>MDBF subscales for good/bad mood; alertness/fatigue; calmness/restlessness pre and post TSST	Significant improvement in task-set shifting (executive function) in treatment group.	y	y	y	y	n
Bosepflug et al	Volunteers in the community	SMC	Fish oil (1.6g eicosapentaenoic acid (EPA) and 0.8g	11	Placebo- corn oil<br><br>24 weeks	10	Working memory task	Significant improvement of working memory in the 2-back	y	y	_	y	n

For peer review only

			docosahexaenoic acid (DHA)) daily.<br><br>24 weeks.					condition. In the treatment group						
Brautigham et al	Volunteers in the community	SCD; non-institutionalised; Age 55-86 years	Gingko 40 drops (1.9mls) undiluted daily<br><br>24 weeks	72	Placebo<br><br>24 weeks	82	15 Words of Rey (part 1 and 2); Benton test of visual retention-revised; EMCT	Statistically significant improvement in visual memory that was dose-dependent	y	y	n	y	n
Brautigham et al	Volunteers in the community	SCD; non-institutionalised; Age 55-86 years	Gingko 40 drops (1.9mls) 1:1 dilution<br><br>24 weeks	72	Placebo<br><br>24 weeks	82	15 Words of Rey (part 1 and 2); Benton test of visual retention-revised; EMCT	Statistically significant improvement in visual memory that was dose-dependent	y	y	n	y	n
Cohen-Mansfield et al	Volunteers in the community	SMC MMSE >24; Hebrew language; Age>65 years	Cogntive training course-participant centred, delivered in a book club format with memory, reasoning and speed of processing being the main foci.	15	Waiting list control	28	UCLA loneliness scale<br><br>Global cognitive score;	Significantly improved global cognitive score and significant reduction in loneliness and self-reported memory difficulties.	y	y	n	y	n

For peer review only

			10 weeks										
Cohen-Mansfield et al	Volunteers in the community	SMC MMSE >24; Hebrew language; Age>65 years	Participant-centered course-delivery of memory, cognitive and organisational strategies. Cognitive behavioural principles used whereby individuals had mistaken conceptions of memory problems and self-worth challenged.<br><br>10 weeks	14	Non-active control	12	UCLA loneliness scale<br><br>Global cognitive score;	Improvement in cognitive scores from pre to post intervention. Trend towards reduction in loneliness. No significant difference compared to control.	y	y	n	y	n
Cohen-Mansfield et al	Volunteers in the community	SMC MMSE >24; Hebrew language; Age>65 years	Health promotion- topics included health behaviours, dementia, cognitive activities, relationships, depression and coping, home and travel safety,	15	Non-active control	12	UCLA loneliness scale<br><br>Global cognitive score;	Improvement in cognitive scores from pre to post intervention. Trend towards reduction in loneliness. No significant difference compared to control.	y	y	n	y	n

For peer review only

			recreation and leisure, medications and health care providers, physical activity and lifelong learning.<br><br>10 weeks										
Hoogenhaut et al	Volunteers in the community	SCD; women; Age 60-75 years	Psychoeducation-information delivered on subjective cognitive difficulties, ageing and internal/external strategies.<br><br>Eight 1.5 hour sessions delivered over 4 weeks.	30	Waiting list control	30	MMI<br><br>Psychological well-being quotient (CES-D, NCQ, SF-36);<br><br>Memory quotient based on VVLT;<br>Executive Functioning and Speed Quotient-(CST, SCWT, LDST)	Participants in the experimental group reported fewer emotional reactions towards cognitive functioning post-intervention.<br><br>No significant differences between groups for psychological well-being, memory or executive function.	y	y	y	y	N

For peer review only

Kwok et al	Volunteers from community centres	SMC; Age >60 years; no physical or psychiatric co-morbidity associated with cognitive impairment; >22 on MMSE	Active mind training programme- education on memory decline and dementia together with cognitive training.<br><br>1 hour, once a week for 8 weeks	86	Active control- usual group activities provided by community centre such as daily discussion about the news, outings and monthly gatherings.	90	Total adjusted CDRS; CDRS scores for attention, Initiation, construction Conceptualisation, SF12-Mental	Significant improvement in mental health subscore on SF 12 questionnaire.<br><br>Significant improvement in overall global cognition with conceptualisation being most marked.	y	y	n	y	Y
Metternich et al	Memory Clinic	Meet criteria for diagnosis of FMD; Age <69; IQ>80;	Psychoeducation, cognitive restructuring, stress management, relaxation and mindfulness techniques.	18	Waiting list control	18	SCL-90 R GSI; PSQ<br><br>MIA	Significant improvement in MSE at 3 month follow up post intervention. No significant difference immediately after the intervention.	y	y	y	y	y
Oh et al	Volunteers in the community	SMC; Smartphone owner; K-MMSE >23	SMART – a smartphone application developed to improve the user's attention and working memory. Tasks have three difficulty levels.	18	Waiting list control	16	MMQ<br><br>CES-D; STAI-S<br><br>MDS	Significant improvement in working memory quotient and auditory-working memory.	y	y	n	y	n

For peer review only

			15-20 minutes daily for 5 days a week for 8 weeks										
Oh et al	Volunteers in the community	SMC; Smartphone owner; K-MMSE >23	Fitbrains- smartphone application providing cognitive training. Three tasks within each session with thirty sessions in total. Recommendation to complete one session daily. Each task was completed twice to increase training time.<br><br>Unclear over what duration training was undertaken but at least 30 days over an 8 week period.	19	Waiting list control	16	MMQ<br><br>CES-D; STAI-S<br><br>MDS	Significant improvement in contentment on MMQ	y	y	n	y	n
Pereira-Morales et al	Volunteers from community centres	MMSE>21; no diagnosis of MCI/ dementia;	Integrated psycho-stimulation programme (IPP):	17	Non-active control- Information brochure on how to reduce risks of	11	SMCQ<br><br>STAI	Significant improvement in metamemory and anxiety in IPP	-	n	-	y	n

For peer review only - http://bmjopen.bmj.com/site/about/guidelines.xhtml

		intact ADLs; no significant co-morbid psychiatric or neurological condition.	Cognitive training, progressive relaxation exercises, meta-memory activities and group discussions to engender socialisation.. 90 mins, 4 times a week for 8 weeks		developing MCI/ dementia		Grober and Buschke short/long term memory; Stroop; WAIS	group compared to baseline.					
Pereira-Morales et al	Volunteers from community centres	MMSE>21; no diagnosis of MCI/ dementia; intact ADLs; no significant co-morbid psychiatric or neurological condition.	Computerised cognitive training (CCT). 60 mins, 4 times a week for 8 weeks.	12	Non-active control- Information brochure on how to reduce risks of developing MCI/ dementia	11	SMCQ STAI Grober and Buschke short/long term memory; Stroop; WAIS	Significant improvement in cognition as compared to baseline.	-	n	-	y	n
*Pike et al	Volunteers from community centres	SMC; Age>60; community dwelling; independent ADLs,; English language	Face-name memory training in an office based setting. Either allocated to receive training based on semantic association (SA)	22 (SA) 25 (SR)	No training	21 21	Cued/uncued recall. MMQ-contentment MCI-memory control beliefs	Spaced retrieval improved recall for cued and uncued names, whilst semantic association improved only recall for cued names. Neither	Y	n	n	n	n

For peer review only

			or spaced retrieval (SR).<br><br>One off 1.5 hour session followed by 2 booster phone calls.					were statistically significant.<br><br>No improvement with either intervention in naturalistic setting when assessed two months later.					
Scogin et al	Volunteers in the community.	SMD; Age > 60;	Self-administered cognitive training from a manual.<br><br>Sixteen one hour study sessions.<br><br>Once weekly phone calls from first author to ensure compliance	20	Waiting list control	27	Memory recall tasks; Benton Visual Retention Test.<br><br>Metamemory Questionnaire.<br><br>Zhung depression scale.	No significant difference in composite measure of metacognition or objective cognitive performance or in depression scores<br><br>No differences in memory complaint scores or depression scores.	n	n	n	y	n
Small et al	Volunteers in the community.	SMI; Age 35-69; absence of severe psychiatric or neurological illness; absence of MCI; not	14-day Healthy lifestyle programme based on mental and physical exercises, healthy diet, and stress reduction techniques.	8	Non-active control.<br><br>Continued lifestyle as usual.<br><br>2 weeks	9	Buschke Fuld Selective Reminding Test; COWAT<br><br>MFQ	Verbal fluency improved significantly in intervention group but no significant difference between groups.	n	n	n	y	n

For peer review only

		taking psychotropic medication	2 weeks					No statistically significant differences between groups.					
Smart et al	Volunteers in the community	SCC; Age 65-85; English language; no significant neurological disease; MMSE ≥25;tolerate MRI;	Mindfulness based therapy<br><br>Eight 2 hour long, once weekly group sessions	8	Active control-psychoeducation<br><br>Five 2 hour long, once weekly group sessions	6	Cognitive complaints index – composite score made from cognitive subscales of GDS, MCQ and MIA<br><br>FFMQ-A	P3 ERP amplitude significantly increased.<br><br>No significant Improvements in self-reported subjective memory complaints, mindful attention measures.	-	y	-	y	n
Tsai et al	Volunteers from community setting.	SMC; Age >55; CDR score 0; Independent ADLs; Chinese language	Cognitive training which included strategies and skills to improve cognitive performance. Also taught meditation and some psychoeducation.<br><br>2 hours, twice weekly for 5 weeks.	14	Active control-cognitive stimulation Which included problem solving exercises and quizzes.<br><br>1.5 hours, once weekly for 8 weeks.	11	ADAS-Cog; MMSE; SRT Clock Drawing Test	Statistically significant improvements in MMSE and ADAS-Cog in both groups at the end of the intervention and 6 months follow up but no significant difference between groups.	n	n	n	y	n

For peer review

Valentijn et al	Volunteers from community setting.	SMC; Age>55; Dutch language; >24 on MMSE; mobile,	Collective memory training including internal and external memory strategies as well as psychoeducation.<br><br>2 hour weekly session for 8 weeks	53	Waiting list control	43	VVLT; Short story test; CFQ<br><br>MIA	Significant improvement in recall.<br><br>Significant improvement on MIA change and anxiety subscale score.	y	n	y	n	n
Valentijn et al	Volunteers in the community	SMC; Age >55; Dutch language; MMSE >24; mobile	Individual training – subjects given "Memory support" book with the content described in the same order as above. 8-week time period (called 4 weeks through to ensure compliance with the training)	43	Waiting list control	43	VVLT; Short story test; CFQ<br><br>MIA	Significant improvement in recall.<br><br>Significant improvement in MIA anxiety scores.	y	n	y	n	n
*Van Hooren et al	Volunteers in the community.	SCC; Age>55; MMSE≥24; Dutch language; travel independently; absence of	Goal management training- structured and interactive intervention to teach individuals	38	Waiting list control	31	SCWT<br><br>CFQ<br><br>SCL-90	Significant reduction in anxiety scores.<br><br>Significant reduction in annoyance towards cognitive failures.	y	n	y	n	n

For peer review only

		psychiatric or neurological co-morbidity.	a strategy to improve planning activities and to structure intentions. Additionally, there was a psychoeducation component about cognitive ageing.<br><br>12 sessions each 1.5 hours long delivered over 6 weeks (1 individual and 11 group sessions)					Significant reduction in number of executive failures reported.						
*Youn et al	Memory clinic	SMC; Age > 55; excluded if serious psychiatric or medical co-morbidity	Cognitive training (focus on metamemory)<br><br>10 week programme, of 90 min 1x weekly sessions	20	Waiting list control	20	SRFT; Elderly verbal learning test; DST; VST; Phonemic fluency test; Categorical fluency test<br><br>SMCQ<br><br>GDS	Significant improvements on short and long term recall, SRFT recognition, VST (forward), categorical fluency.	y	Y	n	y	n	

Zhu et al	Volunteers in the community	SCC; Age ≥50 years, not on psychotropic medication, , no severe psychiatric or medical conditions	Brain Power Advanced supplement.<br><br>2 capsules with meals for 12 weeks.	47	Non-active control- placebo tablets.<br><br>2 capsules with meals for 12 weeks.	51	Visual, auditory and short-term memory<br><br>Subjective memory loss and subjective attentional deficits.	No significant difference between in subjective memory loss or subjective attentional deficit scores.	Y	y	n	y	n

SMC, Subjective memory complaints; MDRS, Mattis Dementia Rating Scale; RAVLT, Rey Auditory Verbal Learning Test; EFT, Eriksen Flanker Test; DSST, Digit Symbol Substitution Test; TMT-A, Trail Making Test A; TMT-B, Trail Making Test B; UFOV, Useful field of view; TSST, Trier Social Stress Test; SMI, subjective memory impairment; PRMQ, Prospective and Retrospective Memory Questionnaire; EMCT, Expanded Mental Control Test; SCD, Subjective Cognitive Decline; MMI, Maastrict Metacognition Inventory; CES-D, Centre of Epidemiologic Studies Depression scale; NCQ, Neurovegetative Complaints Questionnaire; SF-36, Short-Form Health Survey; CST, Concept Shifting Test; SCWT, Stroop Color-Word Test; LDST, Letter Digit Substitution; VVLT, Visual Verbal Learning Test; SF12, 12-Item Short Form Health Survey; CDRS, Chinese version of Mattis Dementia Rating Scale; SCL-90 R GSI, Symptom Checklist 90-R; MIA, Metamemory in Adulthood Questionnaire; PSQ, Perceived Stress Questionnaire; FMD, Functional Memory Disorder; WAIS, Wechsler Adult Intelligence Scale; STAI, State-Trait Anxiety Inventory; SMCQ, Subjective Memory Complaints Questionnaire; MMQ, Multifactorial Memory Questionnaire; MDS, Memory Diagnostic System; MCI, Mild Cognitive Impairment; SRT, Selective Reminding Test; MMSE, Mini-Mental State Examination; CDR, Clinical Dementia Rating; ADAS-cog, Alzheimer's disease Assessment Scale-Cognition; CFQ, Cognitive Failure Questionnaire; SRFT, Simple-Rey figure test; DST, Digit Span Test; VST, Visual Span Test; MCQ, Memory Complaints Questionnaire; FFMQ-A, five facet mindfulness questionnaire- "mindful attention" subscales; COWAT, Controlled oral word association test; MFQ, Memory Function Questionnaire; SMI, Subjective Memory impairment.

***Not included in meta-analysis**

For peer review only

PRISMA 2009 Checklist

Section/topic	#	Checklist item	Reported on page #
**TITLE**			
Title	1	Identify the report as a systematic review, meta-analysis, or both.	1
**ABSTRACT**			
Structured summary	2	Provide a structured summary including, as applicable: background; objectives; data sources; study eligibility criteria, participants, and interventions; study appraisal and synthesis methods; results; limitations; conclusions and implications of key findings; systematic review registration number.	2
**INTRODUCTION**			
Rationale	3	Describe the rationale for the review in the context of what is already known.	4
Objectives	4	Provide an explicit statement of questions being addressed with reference to participants, interventions, comparisons, outcomes, and study design (PICOS).	4
**METHODS**			
Protocol and registration	5	Indicate if a review protocol exists, if and where it can be accessed (e.g., Web address), and, if available, provide registration information including registration number.	1
Eligibility criteria	6	Specify study characteristics (e.g., PICOS, length of follow-up) and report characteristics (e.g., years considered, language, publication status) used as criteria for eligibility, giving rationale.	5
Information sources	7	Describe all information sources (e.g., databases with dates of coverage, contact with study authors to identify additional studies) in the search and date last searched.	5
Search	8	Present full electronic search strategy for at least one database, including any limits used, such that it could be repeated.	5
Study selection	9	State the process for selecting studies (i.e., screening, eligibility, included in systematic review, and, if applicable, included in the meta-analysis).	5
Data collection process	10	Describe method of data extraction from reports (e.g., piloted forms, independently, in duplicate) and any processes for obtaining and confirming data from investigators.	5
Data items	11	List and define all variables for which data were sought (e.g., PICOS, funding sources) and any assumptions and simplifications made.	5
Risk of bias in individual studies	12	Describe methods used for assessing risk of bias of individual studies (including specification of whether this was done at the study or outcome level), and how this information is to be used in any data synthesis.	5, supplementary data files 1 and 2
Summary measures	13	State the principal summary measures (e.g., risk ratio, difference in means).	5, supplementary

For peer review only - http://bmjopen.bmj.com/site/about/guidelines.xhtml

PRISMA 2009 Checklist

			data file 1
Synthesis of results	14	Describe the methods of handling data and combining results of studies, if done, including measures of consistency (e.g., I^2) for each meta-analysis.	5, figures 2-5 supplementary data file 1

Section/topic	#	Checklist item	Reported on page #
Risk of bias across studies	15	Specify any assessment of risk of bias that may affect the cumulative evidence (e.g., publication bias, selective reporting within studies).	5, supplementary files 1 and 2
Additional analyses	16	Describe methods of additional analyses (e.g., sensitivity or subgroup analyses, meta-regression), if done, indicating which were pre-specified.	Not done
**RESULTS**			
Study selection	17	Give numbers of studies screened, assessed for eligibility, and included in the review, with reasons for exclusions at each stage, ideally with a flow diagram.	6, Figure 1
Study characteristics	18	For each study, present characteristics for which data were extracted (e.g., study size, PICOS, follow-up period) and provide the citations.	Supplementary data file 2
Risk of bias within studies	19	Present data on risk of bias of each study and, if available, any outcome level assessment (see item 12).	5, supplementary data files 1 and 2
Results of individual studies	20	For all outcomes considered (benefits or harms), present, for each study: (a) simple summary data for each intervention group (b) effect estimates and confidence intervals, ideally with a forest plot.	6-9, Figures 2-5, supplementary data file 2
Synthesis of results	21	Present results of each meta-analysis done, including confidence intervals and measures of consistency.	Figures 2-5
Risk of bias across studies	22	Present results of any assessment of risk of bias across studies (see Item 15).	Not done
Additional analysis	23	Give results of additional analyses, if done (e.g., sensitivity or subgroup analyses, meta-regression [see Item 16]).	Not done
**DISCUSSION**			
Summary of evidence	24	Summarize the main findings including the strength of evidence for each main outcome; consider their relevance to key groups (e.g., healthcare providers, users, and policy makers).	9-11

For peer review only - http://bmjopen.bmj.com/site/about/guidelines.xhtml

PRISMA 2009 Checklist

Limitations	25	Discuss limitations at study and outcome level (e.g., risk of bias), and at review-level (e.g., incomplete retrieval of identified research, reporting bias).	10-11
Conclusions	26	Provide a general interpretation of the results in the context of other evidence, and implications for future research.	11
**FUNDING**			
Funding	27	Describe sources of funding for the systematic review and other support (e.g., supply of data); role of funders for the systematic review.	12

From: Moher D, Liberati A, Tetzlaff J, Altman DG, The PRISMA Group (2009). Preferred Reporting Items for Systematic Reviews and Meta-Analyses: The PRISMA Statement. PLoS Med 6(7): e1000097. doi:10.1371/journal.pmed1000097

For more information, visit: **www.prisma-statement.org**.

For peer review only

For peer review only - http://bmjopen.bmj.com/site/about/guidelines.xhtml
